# Comparative performance of rural water supplies during drought

**DOI:** 10.1038/s41467-020-14839-3

**Published:** 2020-03-04

**Authors:** D. J. MacAllister, A. M. MacDonald, S. Kebede, S. Godfrey, R. Calow

**Affiliations:** 1 British Geological Survey, The Lyell Centre, Edinburgh, UK; 2 School of Agricultural Earth and Environmental Sciences, University of KwaZulu Natal, Pietermaritzburg, South Africa; 3 UNICEF Regional Office for Eastern and Southern Africa, Nairobi, Kenya; 4 Overseas Development Institute, London, UK

**Keywords:** Hydrology, Climate-change adaptation, Climate-change mitigation, Sustainability, Hydrology

## Abstract

As rural African communities experience more frequent and extreme droughts, it is increasingly important that water supplies are climate resilient. Using a unique temporal dataset we explore rural water supply (*n* = 5196) performance during the 2015–16 drought in Ethiopia. Mean functionality ranged from 60% for motorised boreholes to 75% for hand-pumped boreholes. Real-time monitoring and responsive operation and maintenance led to rapid increases in functionality of hand-pumped and, to a lesser extent, motorised boreholes. Increased demand was placed on motorised boreholes in lowland areas as springs, hand-dug-wells and open sources failed. Most users travelled >1 h to access motorised boreholes but <30 min, increasing to 30-60 mins, for hand-pumped boreholes. Boreholes accessing deep (>30 m) groundwater performed best during the drought. Prioritising access to groundwater via multiple improved sources and a portfolio of technologies, such as hand-pumped and motorised boreholes, supported by responsive and proactive operation and maintenance, increases rural water supply resilience.

## Abstract (web summary)

The authors compared the performance of a range of rural water supply types during drought in Ethiopia. They show that prioritising access to groundwater via multiple improved water sources and technologies, such as hand-pumped and motorised boreholes, supported by monitoring and proactive operation and maintenance increases rural water supply resilience.

## Introduction

As a result of climate change, sub-Saharan Africa is expected to experience more frequent and extreme droughts, contributing to greater water insecurity^1^. Droughts affect the reliability, quantity and quality of water available^2,3^, potentially undermining recent gains in drinking water access and making it more difficult to extend services to vulnerable populations^4,5^. Groundwater is typically a resilient water resource^5–7^, but few studies have directly compared the performance of different rural water supply technologies accessing groundwater during drought. One recent field study compared a small sample of springs, boreholes and hand-dug-wells in rural Africa and found that boreholes equipped with hand-pumps were more resilient than springs and hand-dug-wells^8^.

Ethiopia is highly vulnerable to the impacts of drought^9^. Drought in 2015–16 caused a large scale humanitarian crisis with significant impacts on food and water security. More than 10 million people across six regions were forced to rely on emergency aid^4^. The 2015–16 drought was exacerbated by the development of a strong El-Niño in 2015 leading to a failure of the June–July–August rains. From a meteorological perspective the 2015–16 drought was at least as severe as the drought in 1983–84 and had a return period of 1 in 60 years^10^. Studies of the impact of climate change on future drought suggest that most of Africa will experience significant increases in drought frequency and magnitude^11^, and rapid population growth is expected to lead to an increased number of people exposed to drought^12^. Furthermore, El-Niño events are predicted to double in frequency as a result of climate change^13^ and will have consequences for drought frequency and magnitude across Africa.

During the period of the Millennium Development Goals (MDGs), set by the international community in the year 2000, Ethiopia increased access to rural point source improved water supplies (e.g. protected hand-dug wells, springs and boreholes equipped with hand-pumps) by 22%^14^. Across sub-Saharan Africa, roughly 73% of people rely on point source water supplies, 47% on improved and 26% on unimproved sources (see Table 1 for distinction between improved and unimproved sources)^14^. However, there is evidence that some point source water technologies, such as springs and hand-dug wells, do not deliver reliable services during drought^8^, are vulnerable to contamination^8,15^, and have higher life-cycle costs than alternatives such as hand-pumped or motorised and piped systems^16^. The Sustainable Development Goals (SDGs) superseded the MDGs at the end of 2015 and are more ambitious (see Table 1 for distinction between SDG and MDG water targets), aiming to achieve (under Goal 6.1) Universal and equitable access to safe and affordable drinking water for all. The key indicator selected for Goal 6.1 is based on water access on premises, available when needed, and free from contamination^17^. Although countries are expected to adopt customised targets that incorporate more basic levels of service, the SDGs offer no guidance on how this customisation should take place in different country contexts or in the face of climate change. Therefore, it is important to understand the level of service and resilience of different water source types in order to chart a way forward.

**Table 1:** Millennium Development Goals (MDG) and Sustainable Development Goals (SDG) water service levels, as defined by WHO and UNICEF Joint Monitoring Programme (JMP), and examples of associated water source types^14,64^.

SDG	MDG	Service level definition	Acceptable water source types	Study water source categories
Safely Managed	Improved	Drinking water from an improved water source which is located on premises, available when needed and free from faecal and priority chemical contamination.	Piped water into dwelling Piped water to yard/plot	N/A
Basic		Drinking water from an improved source, provided collection time is not more than 30 min for a roundtrip including queuing.	Public tap or standpipe Tubewell or borehole Protected dug-well Protected spring Rainwater Delivered water (i.e. tankers/carts)	Hand-pumped boreholes Motorised boreholes Protected wells Springs Water trucking
Limited		Drinking water from an improved source for which collection time exceeds 30 min for a roundtrip including queuing.	Public tap or standpipe Tubewell or borehole Protected dug well Protected spring Rainwater Delivered water (i.e. tankers/carts)	Hand-pumped boreholes Motorised boreholes Protected wells Springs Water trucking
Unimproved	Unimproved	Drinking water from an unprotected dug-well or unprotected spring.	Unprotected dug-well Unprotected springs	Open sources
Surface water		Drinking water directly from a river, dam, lake, pond, stream, canal or irrigation canal.	Untreated surface water sources	Open sources

The relationship between the water source categories used in this study and the MDG and SDG definitions is also shown.

**Fig. 1:**
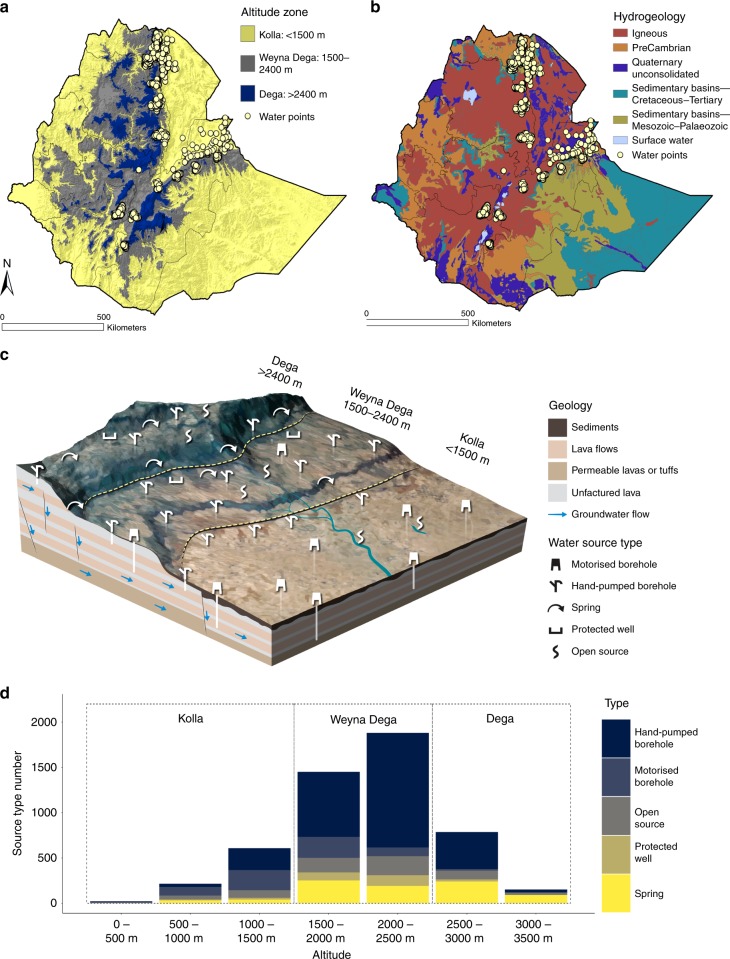
Location and number of water sources, altitude zones and hydrogeology of the study area. **a** Altitude zones of Ethiopia and locations of water points monitored during the survey period. **b** Hydrogeology of Ethiopia^61^. **c** Basic conceptual model of hydrogeology and rural water supply provision in Ethiopia. **d** Number of each water source type with altitude.

**Fig. 2:**
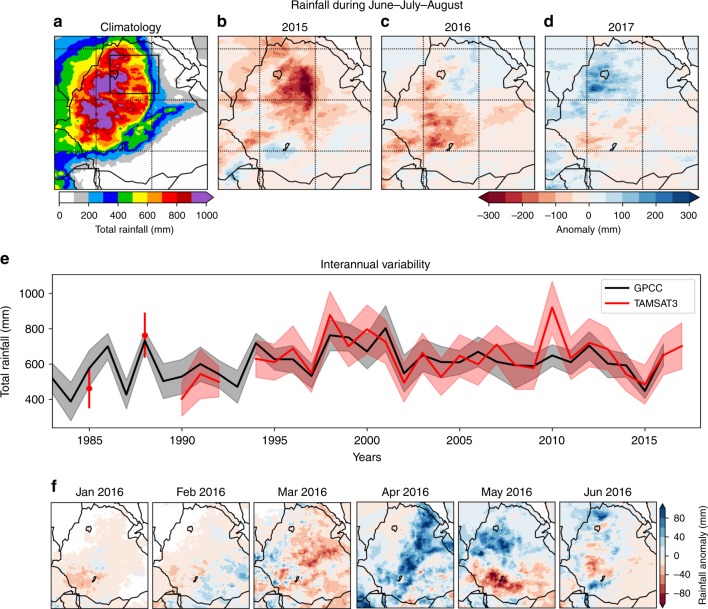
Rainfall during the drought monitoring and intervention period. Summary of total rainfall during June–July–August (JJA) rains. **a** 1983–2013 JJA climatology. **b**–**d** 2015, 2016 and 2017 JJA anomalies relative to **a**. **e** Interannual variability in JJA total rainfall over the study area from TAMSAT and the station-based GPCC Full Data Reanalysis version 8^62^, the solid lines represent the regional mean while the shading represents the regional standard deviation. **f** Rainfall anomaly for the period of the monitoring survey relative to **a**. Rainfall data is estimated from the University of Reading’s Tropical Applications of Meteorology using SATellite data and ground-based observations (TAMSAT^©^) product^63^. **a**–**e** is adapted from MacDonald et al.^8^. Maps are based on data from the digital chart of the World (ESRI).

Here we analyse the performance of a wide range of water source types in Ethiopia (Fig. 1), using a unique dataset of more than 5000 individual water points^18^. Monitoring was conducted weekly during the worst of the drought (Fig. 2) and is described in more detail in the Methods. Details on the numbers of sites and source types visited during the monitoring survey can be found in Supplementary Figs. 1–10. Using this dataset, the aim of our study was to examine the performance of boreholes equipped with hand-pumps (hand-pumps), motorised boreholes, protected springs (springs), protected hand-dug-wells (protected wells) and open sources such as unprotected hand-dug-wells and surface water sources. These source types are the most commonly used in rural Ethiopia and across much of sub-Saharan Africa. Each source type typically draws water from different depths and potentially from different hydrogeological zones (Fig. 1d), needs different levels of external support and has different operation and maintenance requirements (see Methods and Supplementary Note 1). Figure 1 shows the location of the water sources included within the monitoring programme along with altitude (Fig. 1a) and hydrogeology (Fig. 1b, c). Altitude is a key driver of precipitation, water availability and access in Ethiopia^6^ so is used here to contextualise the performance of the different water source types (Fig. 1c, d) monitored during the drought. Figure 2 shows the long-term climatology and rainfall anomalies prior to and during the monitoring period. Table 1 illustrates the relationship between MDG and SDG service level targets, the difference between improved and unimproved sources, and the water source type categories used in our study. Water source performance was evaluated using source functionality (see Methods for definition), user numbers, travel times for water collection and source user perceptions of water quantities collected.

Our results show that hand-pumped and motorised boreholes accessing deep (>30 m) groundwater performed best during the drought. Furthermore, rural water supply resilience was increased by ensuring continuous access to groundwater via multiple improved sources and a portfolio of technologies during the drought. Hand-pumped and motorised boreholes experienced large increases in functionality during the drought due to real-time monitoring and responsive and proactive operation and maintenance.

## Results

### Functionality

**Fig. 3:**
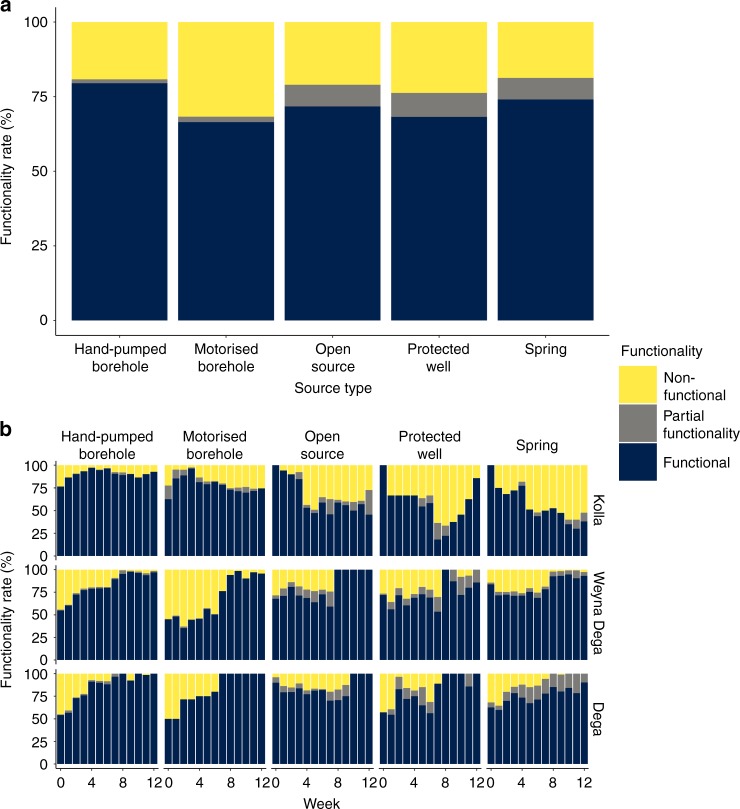
Water source functionality. **a** Overall functionality of each water source type during the drought. Supplementary Figure 2 shows the number of site visits and for which functionality data are available. **b** Weekly functionality rates of each water source type in the Ethiopia’s three altitude zones. Supplementary Figure 5 provides details on the numbers of each source type visited in each altitude zone and for which functionality data are available.

Figure 3a shows overall levels of functionality for the duration of the monitoring survey, hand-pumps had the highest overall functionality and motorised boreholes the lowest. Figure 3b shows the evolution of functionality in each altitude zone during the monitoring period (see Supplementary Figs. 1–6 for source type numbers used to derive functionality rates, Supplementary Fig. 11 for overall water source type functionality per week, Supplementary Fig. 12 for functionality per administrative region, and Supplementary Fig. 13 for functionality of water sources within 500 m altitude bins). Springs, open sources and protected wells experienced large declines in functionality during the monitoring period in the lowest areas of the Kolla. Motorised boreholes experienced an initial increase in functionality and then more gradual declines within the Kolla. Hand-pumps experienced a similar initial increase in functionality in the Kolla but after week 8 functionality remained approximately constant for the rest of the monitoring period. In the highland areas, of the Dega and Weyna Dega, functionality increased for all source types with most significant increases in functionality for motorised boreholes from week 5 onwards. Increases in functionality of hand-pumped and motorised boreholes was primarily due to the initiation of an intensive programme of preventative operation and maintenance, started at the same time as the monitoring survey. Increases in functionality of open sources, protected wells and springs, is more likely to be related to the increases in rainfall from April 2016 onwards (Fig. 2). Functionality also varied by administrative area (Supplementary Figs. 4 and 12).

### Source usage

**Fig. 4:**
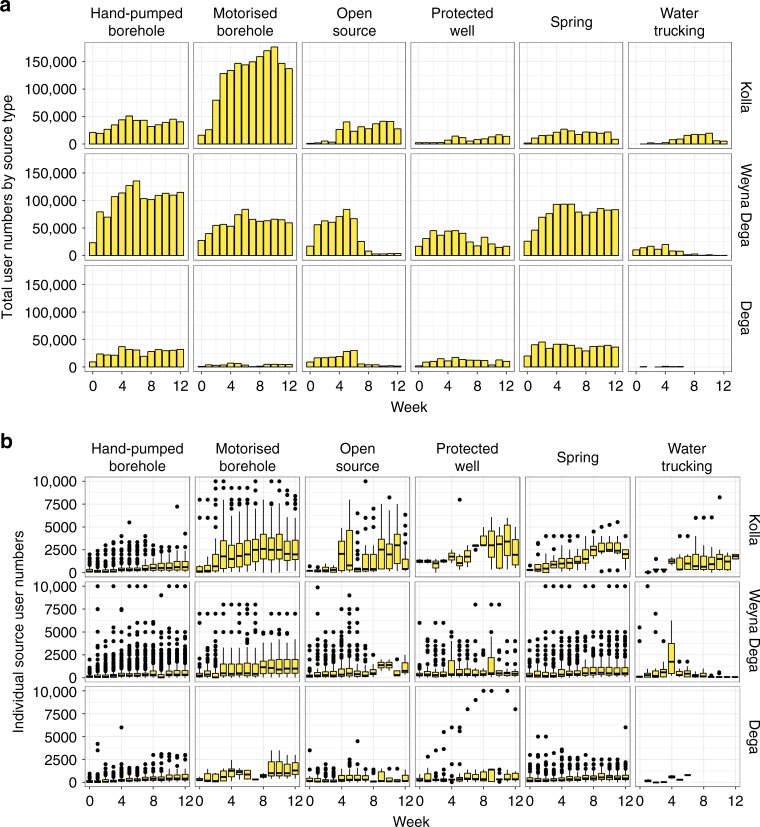
Reported user numbers by water source type. **a** Total user numbers for each water source type in Ethiopia’s three altitude zones. **b** User numbers for individual sources in each of Ethiopia’s three main altitude zones for each water source type. The interquartile range is shown by the upper and lower bounds of the yellow box, the median is represented by the black line in the yellow box, the whiskers represent the interquartile range times 1.5 and the points represent outliers. Supplementary Figure 7 provides details on the numbers of each source type visited in each region and for which estimated user numbers are available.

Figure 4 shows the evolution of usage by water source type during the drought and includes the number of users relying on emergency water trucking. Approximately 830,000 people relied on the water sources included in the monitoring survey, representing ~25% of the overall population of the monitoring areas (Supplementary Table 1).

Motorised and hand-pumped boreholes served the largest number of people (Fig. 4a), providing water to ~400,000 people or roughly 50% of the population covered by the survey. Motorised boreholes served more than 150,000 people in the Kolla, while hand-pumps served a similar number in the Weyna Dega. Springs were a very important water source for ~150,000 people in the Weyna Dega and Dega throughout the drought. Approximately 50,000 people were provided with water via emergency water trucking during the survey period. The numbers collecting from water trucks declined in the Weyna Dega as shallow groundwater and surface water sources recovered from week 8 onwards, but increased in the Kolla as the functionality of springs, protected wells and open sources declined (Fig. 3b).

All individual water sources in the Kolla experienced significant increases in user numbers (Fig. 4b) during the monitoring period. The median population served by individual water sources, of all types, exceeded 1000 people from week 4 onwards, despite declines in functionality of shallow groundwater and surface water sources (Fig. 3b). However, hand-pumps and motorised boreholes, which accessed deep (>30 m) groundwater, experienced the most regular increase in user numbers. By week 4 motorised boreholes had median user numbers of ~2500 people. Thus, deep groundwater provided a buffer for communities as shallow groundwater and surface water sources failed. However, it is clear that in all areas many communities continued to access more vulnerable groundwater and surface water sources, despite significant declines in functionality, particularly in the Kolla.

### Water access

**Fig. 5:**
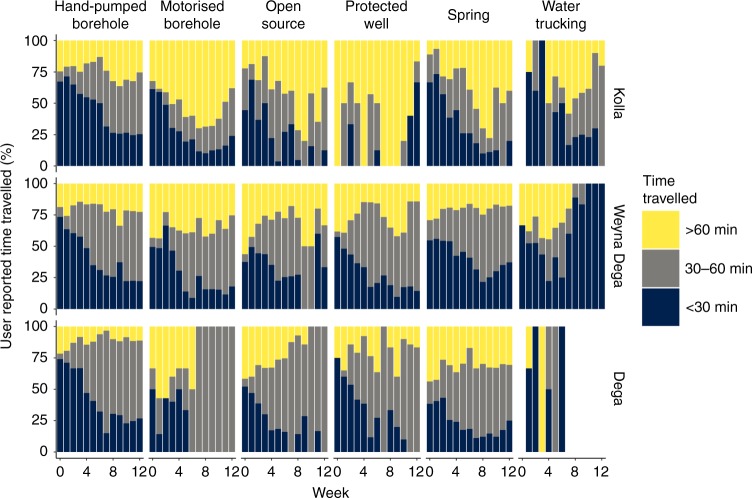
Reported travel times by water source type. Travel times to access water in each of Ethiopia’s three main altitude zones for each water source type. Supplementary Figure 8 provides details on the numbers of each source type visited in each region and for which estimated collection times are available. Note that there were only three sites that were reliant on water trucking in the Dega during the monitoring period, these sites were not monitored consistently leading to gaps in the time series.

Figure 5 shows the evolution of user reported travel times for water collection for each source type during the monitoring period. For all source types, and in all areas, there was a general increase in the amount of time spent collecting water. Travel times were highest overall in the Kolla and lowest in the Dega. In the Kolla most users of motorised boreholes travelled for over 60 min to access water. Only around 25% of hand-pump users in the Kolla travelled for more than 60 min. For hand-pumps in the Dega and motorised boreholes in the Weyna Dega, the number of users travelling for more than 60 min decreased as the monitoring period progressed. Those collecting water from springs spent less time travelling from week 8 of the monitoring period as these source recovered (Fig. 3b) due to increased rainfall (Fig. 2f) in April.

**Fig. 6:**
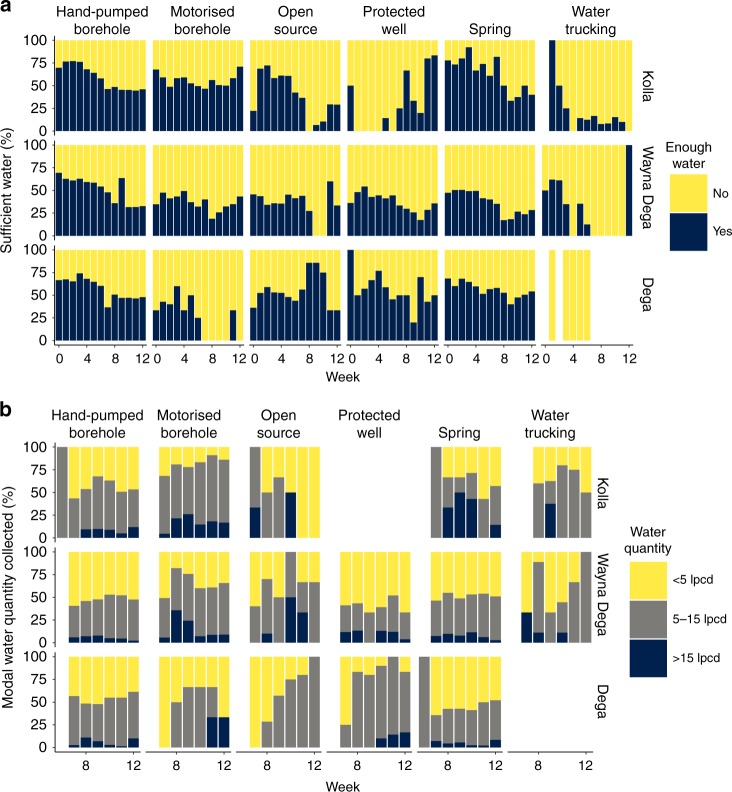
Reported perceptions of water quantity by water source type. **a** Users perception of water quantity and **b** reported water quantities in litres per capita per day (lpcd), based on a household survey conducted from week 7 to the end of the monitoring period, in Ethiopia’s three main altitude zones for each water source type. Supplementary Figures 9 and 10 provide details on the numbers of each source type visited in each region and for which water quantity data is available.

Figure 6a shows user perceptions of the adequacy of water quantities collected during the drought. In all cases more users reported not having enough water as the monitoring period progressed. The largest change in perceptions of adequate quantity was for springs in the Kolla, with a decline of ~50% of users reporting enough water. Approximately 50% of motorised borehole users reported collecting adequate quantities of water, a proportion that did not change significantly during the drought. Approximately 70% of hand-pump users reported collecting an adequate quantity of water at the start of the monitoring period but this declined to around 50% by the end of the monitoring period. In the Kolla the majority (>50%) of users reported collecting adequate quantities of water from springs, hand-pumps and motorised boreholes. In the Weyna Dega most users reported having inadequate quantities of water. In the Dega most users reported having adequate quantities from most sources, except from the small number of motorised boreholes. Those that relied on water trucking reported the lowest perceptions of adequate water quantity. Figure 6b show results from a household survey that was conducted in the last five weeks of the monitoring period, from late March to the early May. The majority of users collected <15 litres per capita per day. Users in the Weyna Dega generally reported collecting the least amount of water. As the drought progressed users in the Kolla reported collecting more water from motorised boreholes and less from open sources. Users in the Dega reported collecting more water from open sources and protected wells later in the drought. For the remaining sources, user reports of water quantities collected remained reasonably constant throughout the drought.

## Discussion

Our results show significant variation in functionality and service level for different water source types. Hand-pumps had highest overall functionality during the monitoring period (c.75%, Fig. 3a) and motorised boreholes the lowest (c.60%, Fig. 3a). In the context of recent debates about the functionality of hand-pumps^8,19–26^ this may be a surprising finding, although high levels of hand-pump functionality are in fact consistent with numerous previous findings in semi-arid areas where hand-pumped boreholes have been found to perform well during drought^2,5,6,8,27^.

It is notable that at the start of the monitoring period hand-pump functionality is higher than motorised borehole functionality in the Kolla regions, and remains consistently higher throughout the drought. At the end of the monitoring period hand-pump functionality in the Kolla is ~15–20% higher than that of motorised boreholes. Hand-pumps do not experience the large declines in functionality experienced by open sources, protected wells and springs (Fig. 3b), suggesting that hand-pump sources access relatively deep (>30 m) groundwater and are a resilient source of water supply during drought.

While motorised boreholes generally also access deeper groundwater, repairs are more difficult^6^ and may take longer, resulting in lower levels of functionality as compared to hand-pumps. Klug et al.^28^ suggest that maintenance and repair of rural water supplies is hindered by limited access to skilled expertise which is often costly and located far from communities. Limitations on access to skilled expertise, spare parts and fuel may increase the downtime of motorised boreholes, particularly when demand for external support increases during drought. Overall levels of functionality also vary by administrative area (see Supplementary Figs. 4 and 12), likely due to differences in capacity and availability of skills. In the Weyna Dega and Dega areas, hand-pumps and motorised boreholes have similar initial functionality (c. 50%), but the increase in functionality is more rapid for hand-pumps (Fig. 3b), again suggesting more rapid repair times as compared to motorised boreholes.

**Fig. 7:**
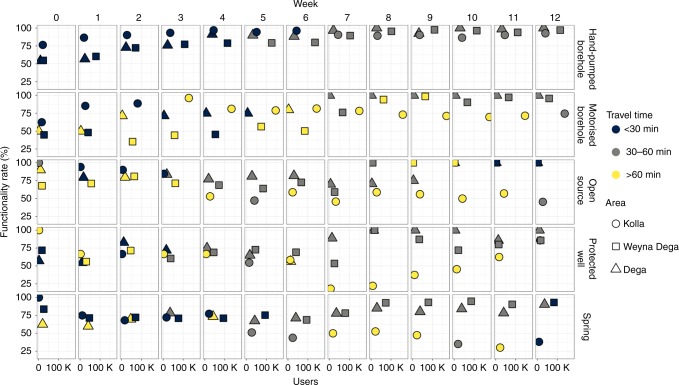
Summary of functionality, user numbers and travel times by water source type and altitude zone. The position of the points in the individual panels represent the functionality and overall user numbers for each water source type (a total of ~830,000 people relied on the water sources included in the monitoring programme), colours represent modal travel times for water collection and shapes represent altitude areas. The evolution of the performance of each water source type (in terms of functionality, access and usage) reveals five key points. (1). Hand-pumped boreholes experienced the most consistent overall increases in functionality in all areas and most users travelled for <60 min for water collection (in the first 6 weeks <30 min). (2) Motorised borehole performance was more variable with very low functionality in the Weyna Dega (<50%) and many users having to travel for >60 min to access water throughout the intervention period. (3) In general hand-pumped and motorised boreholes, which accessed deep groundwater (>30 m), were a very important water source for many people during the drought and provided water to ~400,000 people; hand-pumps provided for >100,000 people in the Weyna Dega. Motorised boreholes provided for ~150,000 people in the Kolla. (4) Springs performed reasonably well in the Weyna Dega and Dega areas providing water (travel times < 60 min) to ~100,000 people in the Weyna Dega. Springs were also an important and accessible form of water supply to Kolla communities early in the drought but experienced large declines in functionality from week 6 onwards. (5) Open sources and protected wells experienced large declines in functionality in the Kolla areas but increased functionality in the Weyna Dega and Dega towards the end of the drought period associated with local rainfall.

Figure 7 summarises the performance, pressures and service provided by the different source types in the three altitude zones of Ethiopia during the drought. Figure 7 illustrates a number of key points. Firstly, it is clear that the motorised boreholes and hand-pumps included within the monitoring programme supplied the majority of people during the drought. From a peak in week five of more than 830,000 users included in the monitoring survey, ~55% rely on motorised and hand-pumped boreholes. Springs, open sources and protected wells, which provide water to the remaining 45% of users, are an extremely important source of water for many. Springs performed reasonably well throughout the drought in Weyna Dega and Dega areas (Fig. 3) and were associated with relatively low modal travel times of 30–60 min for water collection (Fig. 6).

Figure 7 also clearly illustrates that Kolla areas are particularly vulnerable to the effects of drought. Here, large numbers of people rely on shallow groundwater and surface water sources (Figs. 4 and 7), although the health risks associated with inadequate water quality and quantity from these source types is high^8,15,5^. With the exception of hand-pumps travel times for water collection are generally longest in the Kolla. However, the percentage of users reporting collecting inadequate quantities of water is not significantly higher than in the other areas. This may be explained by the fact that communities in the Kolla are predominantly pastoral and agro-pastoral and tend to be more mobile, having access to the animals needed to transport water^29^. Our results indicate that drought-induced water scarcity is a fact of life for many in the Kolla but that communities have learned to adapt.

In general the drought did not lead to large scale failure of deep (>30 m) groundwater sources, such as hand-pumped and motorised boreholes (Fig. 7). Rather drought led to changes in demand, access and perceptions of the adequacy of water quantities collected, confirming previous studies that suggest these factors are more important than physical water scarcity in determining household water security^6,30^. However, the drought did lead to a large scale decline in the performance of surface and shallow groundwater sources (springs, open sources and protected wells), particularly in the Kolla areas, thus undermining the water security of lowland households relying on these sources. The decline in performance of these sources may explain why, in a multi-country comparison of water service continuity, DuChanois et al.^30^ found that only in Ethiopia, where three quarters of their sample consisted of springs and open sources, did drought and water scarcity correlate with declines in water source performance. However, springs, protected wells and open sources begin to recover towards the end of the monitoring period (due to increased rainfall in April shown in Fig. 2f).

Our analysis has a number of implications for resilient rural water supply. Firstly, we note that the increased number of users arising as a result of the drought is spread across different sources and technology types (Figs. 4 and 7). By spreading increased demand across a more diverse range of technologies and a higher number of water sources (i.e. the availability of a portfolio of water supply options) overall service levels are arguably higher than they would be otherwise. As a result, the vast majority of people continued to access water using existing sources and without relying on emergency water trucking. The effectiveness of community access to multiple water sources is emphasised by a number of other studies, which highlight the adaptive capacity^31^ and resilience^32^ inherent in the availability of a portfolio of water supply options at the community level. Furthermore, there is an emerging body of evidence which demonstrates that individual households draw on a range of water sources and technology types in response to variations in rainfall, water availability and quality, and source performance, in order to meet an array of needs, including drinking, cooking, personal hygiene and care of livestock^8,31,33^.

In many areas it may be beneficial to facilitate and formalise a portfolio approach to rural water supply, recognising that in many communities such a practice already occurs and appears to be an effective means of increasing resilience of rural communities^32^. Formalisation of a portfolio approach would include facilitating the ability of communities to access a variety of improved water sources, using different technologies and providing different service levels tailored to different needs, uses and environments, to ensure some for all rather than more for some water supply outcomes. This would require investment in operation and maintenance of existing improved water sources and the commissioning, construction and operation and maintenance of new sources particularly where communities rely on unimproved sources such as surface water or unprotected hand-dug-wells. Our results demonstrate that the availability of a portfolio of improved water supply options is more likely to ensure continued access to water during drought, thereby increasing resilience^32^ and reducing the risk of people returning to unimproved sources^33^.

However, a portfolio approach to rural water supply may not be easily accommodated by the current international agenda. Communal improved point source water supplies, such as hand-pumps and motorised boreholes with standpipes, are not classified as safely managed drinking water supplies under SDG Goal 6.1 (see Table 1) which may discourage the adoption of a portfolio approach, which uses these source types, in national policies. Safely managed water supplies which provide on-plot access to water, for example through piped supply, have many potential benefits. However, emphasising on-plot access, at the expense of communal water sources, is also likely to increase the complexity and cost of services, and leave many people unserved where budgets are stretched. It may also discourage investment in more simple technologies (such as hand-pumps) which commonly form the basis of a community water supply portfolio^31^ and which, we demonstrate, are a resilient form of water supply during drought.

Our results highlight two key reasons for continued investment in improved point source water supplies. First we clearly demonstrate that hand-pumps are crucial to maintaining access to water during drought in the highland (Weyna Dega and Dega) areas of Ethiopia, and to a lesser extent in the lowlands (Kolla). Figure 7 shows that hand-pumped borehole functionality is high and that repairs are conducted quickly during the drought intervention period. It also illustrates that in general hand-pumped boreholes were associated with the shortest travel times for water collection during the drought. Thus, hand-pumped boreholes, although providing a basic service by SDG definitions (see Table 1), provide the most consistent and sustained levels of access to water supply for rural populations affected by the drought. More complex technologies, including on-plot piped water supplies, may struggle to maintain functionality levels sufficient to ensure adequate and sustained access to water during drought, particularly in areas where it is more challenging to find adequate skills and sufficient levels of external support necessary for effective operation and maintenance. Second, communities in more water scarce, vulnerable and inaccessible areas, may be left behind as investment gravitates to piped schemes requiring more productive water sources and skilled management. Furthermore, existing evidence suggests that poor households have lower rates of access to piped water supplies than higher income households^34^. Such households and/or communities may be forced to rely on hand-dug-wells, springs and surface water sources that offer little resilience to drought, and are vulnerable to contamination^8,15,35^.

The most appropriate technical intervention in a given area is determined by hydrological, hydrogeological and demographic constraints. For example in some areas of the Kolla groundwater levels can be up to 250 m deep^36–38^, well beyond the maximum capacity of a hand-pump (typically 45 m). In these areas motorised boreholes are the only appropriate technological solution. In some areas, piped water supply, possibly supplied by springs (which performed reasonably well in some highland areas), deep groundwater or treated surface water, may be a feasible solution. In others, dispersed populations may limit the economic feasibility of more complex piped schemes^39^. Further research is required to understand the technical, physical, social and economic feasibility of such schemes in Ethiopia and across sub-Saharan Africa.

The importance of responsive and proactive operation and maintenance of rural water supplies is illustrated by the rapid increase in, and then sustained levels, of functionality of hand-pumps during the drought (Figs. 3b and  7). Lower, and less rapid increases in functionality, of motorised boreholes, suggest that more investment is required in the skills base and supply chains needed to maintain these more complex systems^28^ that were essential for ensuring continued access to water for many communities in the lowlands (Kolla) during the drought. The rapid increases in functionality that occurred illustrate that responsive and proactive operation and maintenance, which was provided by mobile maintenance teams during the drought (see Supplementary Note 2), reduced the need for costly investments in new infrastructure as an emergency drought response measure. Previous work has shown that investment in new infrastructure, such as the construction of new boreholes, is not an effective means of meeting immediate water needs during drought^6^. Rather, ongoing investment in operation and maintenance not only ensures that overall levels of functionality are higher in general, but also that life-cycle costs are lower, since the need for costly rehabilitation is less frequent^40^. Furthermore, targeted investment in operation and maintenance of technologies capable of accessing deep (>30 m) groundwater sources which are less likely to fail during drought, such as hand-pumps and motorised boreholes, will reduce life-cycle costs further by decreasing the need for investment in expensive emergency responses^16^.

Our analysis raises concerns over the baseline (i.e. pre-drought intervention) functionality of both hand-pumps and motorised boreholes. Low functionality of rural water supplies is in agreement with the findings of a number of studies of hand-pumps^20–26^ and other rural water supply types^41,42^ in sub-Saharan Africa. However, the large increases in functionality that occurred during the drought clearly illustrate the value, and effectiveness, of responsive and proactive operation and maintenance provided by skilled technicians external to communities. Thus, our analysis provides further evidence of the limits of community management alone for ensuring sustainable and resilient water supplies^43^. External support can be provided in different ways with potential roles for government, civil society and the private sector increasingly discussed in the literature^44–48^.

By improving operation and maintenance provision during normal periods, increased demand on hand-pumps and motorised boreholes at the onset of drought will be spread across a larger number of functioning water supplies. This, in turn, will help reduce pressures on individual water sources. Reduced pressures on individual water sources should then result in higher overall levels of functionality and better continuity of water supply during drought.

Real time monitoring, swift data transfer and the consequential availability of decision relevant-information facilitated rapid response to the impacts of the 2015-16 drought in Ethiopia. Our analysis reveals that this near real time monitoring had some success, with functionality increasing approximately in line with increases in user numbers for hand-pumps (Fig. 7). The relationship is not as clear for motorised boreholes, but that may be due to lag times between breakdowns and access to the necessary skills required to repair more complex technologies.

The monitoring programme involved a large number of enumerators spending around 16 weeks continually travelling between water points. Although the success of the programme was facilitated by the use of a mobile based survey tool, which helped streamline the data collection and analysis^49^, it is unlikely that such high intensity monitoring programmes are economically or practically feasible in normal conditions. Recently, however, there have been a number of innovations in smart monitoring technologies, using sensors on hand-pumps^31,50–53^ and motorised boreholes^54^. Such technologies not only allow real time and on-going monitoring of functionality, facilitating more responsive and proactive operation and maintenance in normal and drought conditions, but also provide data on usage patterns of individual water sources and lengths of downtime. Such technologies may help inform future interventions ahead of and during drought.

A significant success of the monitoring programme was the ability to rapidly turn raw data into decision-relevant information. The data were subject to basic automated analysis and sent to centralised servers in near real time, allowing rapid responses to changing water source and service conditions over time. Recent work shows that where service providers (whether public, private or NGO) design streamlined fault reporting systems, such as those facilitated by smart sensors or mobile survey tools, accountability and responsiveness increase^55^. Furthermore, innovative information and communication technologies appear to have the greatest impact when coupled with a responsive operation and maintenance model^55^. A service delivery model that accounts for the social, technical and programmatic aspects of monitoring, that is adaptive, and which clearly establishes how data and information should flow and be used, is most likely to be successful^56^. Our study demonstrates that clear fault reporting mechanisms, paired with responsive and proactive operation and maintenance, can successfully ensure continued access to water during drought in a rural African context.

In summary, the 2015-16 drought in Ethiopia had significant impacts on access to water. Using a unique high-resolution temporal dataset of water source functionality, usage and access we conducted a comparative analysis of the performance of different water supply technologies using a dataset of several thousand water sources in highland and lowland areas of Ethiopia. Such an analysis is unique in that most studies of functionality, whether in drought or normal conditions, rely on single snapshot assessments of water source performance and/or are conducted on a very small sample of water points. We find all sources are put under considerable strain during drought but that most demand was placed on motorised boreholes in lowland areas. While users report having to travel for longer to access water from motorised boreholes, perceptions of sufficient quantity of water remain consistent for motorised sources but declined for most other sources during the drought. Increases in functionality for motorised boreholes lagged behind those of hand-pumped boreholes, suggesting that more complex technologies have longer downtimes, possibly due to a lack of appropriate and/or accessible skills for operation and maintenance. Investment in motorised systems, and the skills to maintain them, is important for ensuring safe and adequate water supply during drought, particularly in areas where groundwater is deep such as the Ethiopian lowlands. However, investment in other sources accessing groundwater, such as hand-pumped boreholes and reliable springs in highland areas, can significantly enhance the resilience of rural water supply during drought. Furthermore, real time-monitoring and effective flow of information to decision makers helps facilitate responsive and proactive operation and maintenance of infrastructure, and ensures that demand is spread across a larger infrastructure portfolio. This, in turn, reduces pressure on rural water supply at the onset, and during, drought.

In conclusion, our analysis clearly demonstrates the value of community access to multiple improved water sources, such as hand-pumped and motorised boreholes, real-time monitoring and responsive and proactive operation and maintenance of those sources. Although political economies differ, the broad lessons of our analysis are relevant across sub-Saharan Africa, where climate change will likely lead to more frequent, longer and more extreme droughts, and where rural communities remain reliant on community managed point source water supplies. We demonstrate that access to multiple sources, adequately monitored and maintained with the assistance of appropriate external support, is a highly effective strategy for sustaining access to water supply, and increasing community resilience, during drought.

## Methods

### Water source types

The monitoring survey and analysis focussed on five main water source types: boreholes equipped with hand-pumps (referred to as hand-pumps, Supplementary Fig. 14); motorised boreholes (Supplementary Fig. 15); protected springs and springs providing gravity fed supplies (referred to as springs, see Supplementary Fig. 16); protected hand-dug-wells (referred to as protected wells, see Supplementary Fig. 17); and open sources (such as unprotected wells and surface water, see Supplementary Fig. 18). During the monitoring period emergency water trucking was also initiated in several areas and is included in the access and usage analysis (Supplementary Fig. 19). These water source types represent the most common technologies used to provide water to rural communities in Ethiopia and across much of sub-Saharan Africa^57^ and, therefore, provide the classification adopted in this paper. More details on each water source type can be found in Supplementary Note 1.

Each source type typically draws water from different depths, and potentially from different hydrogeological zones. In terms of depth, the following definitions are used: shallow groundwater (0–15 m accessed via hand-dug-wells, springs and open sources); intermediate groundwater (15–30 m accessed via hand-dug-wells and hand-pumps); deep groundwater (>30 m accessed via hand-pumps and motorised boreholes); and surface water (open sources). Figure 1d shows a conceptual model of rural water supply provision in Ethiopia. In addition to drawing from a slightly different resource base each source requires different types and levels of operation and maintenance and external support^28^. Brikké and Bredero^57^ provide a comprehensive overview of the different water source types and technologies found in the context of rural water supply in sub-Saharan Africa.

### Water source monitoring

Water source monitoring data were collected from January to May 2016 by teams commissioned by UNICEF following the severe drought in Ethiopia in late 2015 and early 2016. The monitoring programme was conducted in three phases and collected data on source functionality, usage and access. Figure 1a shows the locations of individual water points surveyed during the monitoring programme. The first phase of the programme comprised of a baseline inventory survey, involving a census-style asset tagging exercise of all water points in the target Woredas (administrative divisions’ equivalent to districts). Details of the Woredas included in the surveys can be found in the supplementary information (Supplementary Table 1). During the census phase, 5196 individual sites were visited. The second phase consisted of a monitoring survey performed weekly on a sub-sample of water points. A total of 3188 sites were visited more than once during the monitoring survey. The mean number of visits per site for the 12 weeks of the survey was 3.5. The total numbers of each water source type visited each week and the total number of sites visited per week in each administrative region are shown in Supplementary Figs. 3 and 4 respectively. The final phase, which was only conducted for the last month of the monitoring programme, consisted of a household survey to establish the quantities of water collected by users during the drought.

For each phase trained enumerators used questionnaires to gather quantitative and qualitative data. Surveys were conducted by questioning water point attendants, a water, sanitation and hygiene (WASH) committee member or another knowledgeable local authority. The surveys were conducted using a mobile monitoring platform called Akvo Flow which is developed by The Akvo Foundation. The Akvo Flow application was installed on Samsung Galaxy J1 smartphones (model number SM-J100H) which used Android version 4.4.4. Digital survey forms were pre-loaded onto the app. All data were stored on the smartphones and automatically sent to the Akvo database whenever an internet connection was available.

### Definitions of functionality

Ideally when assessing functionality a clear definition separate from the users experience of the water source should be used. For example, Bonsor et al.^58^ used a tiered approach to hand-pumped borehole functionality, where functionality includes details of hand-pump reliability and yield^58^. In the UNICEF commissioned survey on which our study was based and which proceeded the work done by Bonsor et al.^40^, three categories of functionality were used: functional, partial functionality and non-functional. These categories were based on users’ response to direct questions about functionality rather than measurements of yield or questions about water source reliability (such as pump downtime in the past year). Thus, the functional category does not necessarily mean that a water source is delivering against its original design yield, but rather that a user perceives a water source to be functioning properly. Similarly, the partial functionality category relates to a user’s perception that a water source is not functioning as usual but still delivers some water. Finally, the non-functional category means that users have reported that no water was available from a particular water source at the time of the monitoring visit. Enumerators performed a basic visual check to confirm these categorisations at the time of each monitoring visit.

### Data analysis

The data was downloaded from Akvo databases, cleaned, checked and analysed. First, changes in functionality during the monitoring period were assessed. Functionality was calculated as a weekly average based on the number of water points surveyed. Source usage was also assessed as a weekly average of user numbers for each individual water point. Finally, water access was analysed based on the most commonly reported travel times for each water source type each week. User perceptions of adequate water quantity and reported water quantities were also assessed based on the most commonly reported responses for each water source type per week. Perceptions of adequate water quantity were assessed by asking the participants to answer yes or no to whether they thought they had enough water. Reported volumes of water collected were only available for the last month of the survey which was when the household survey was conducted.

Precipitation in Ethiopia, shown in Fig. 2 for the duration of the monitoring period, is highly complex, but a significant controlling factor is topography^59^. By convention Ethiopia is split into three main agroecological zones based on differences in climate and agricultural practice with altitude^60^. Figure 1a shows the spatial extent and altitude of these three zones. For simplicity, we use the term altitude zone instead of agroecological zone. Due to the influence altitude has on rainfall within Ethiopia we use the three altitude zones as a framework to analyse the comparative performance of different water technologies during the drought (Fig. 1c) and to assess in what areas water supplies and users experienced most severe pressure. Calow et al.^6^ and Tucker et al.^29^ previously described differences in water availability, access and use within these altitude zones, providing support for our choice of framework.

The lowest zone is called the Kolla and is characterised by significant rainfall variability, high temperatures and recurring drought. The Kolla has a large groundwater resource base, including very deep groundwater, with relatively low human population and low demand on water resources. There is relatively higher demand on water resources from livestock in the Kolla. The lower part of the Kolla, below 500 metres, is known as the Berha and is characterised by a lack of rainfed agriculture, limited shallow groundwater and persistent drought. Above the Kolla is the Weyna Dega which is characterised by rainfed agriculture and at least one cropping season a year. The Weyna Dega has a moderate water resource base, but with the largest population water supplies experience high demand from people and livestock. The Dega is the highest zone and has the lowest temperatures. The Dega is characterised by rainfed agriculture but has less crop variability than the Weyna Dega. The Dega has a small water resource base but with lower demand on water supplies. Water is primarily used for household consumption. The area above 3800 metres is sometimes known as the Wurch. For the purposes of the analysis reported here and due to the low number of water sources in the Wurch and the Berha these areas were included within the Dega and Kolla areas respectively. The number of sites visited in each altitude zone and each 500 m altitude bin per week are shown in Supplementary Figs. 5 and 6 respectively.

### Reporting summary

Further information on research design is available in the Nature Research Reporting Summary linked to this article.

## Supplementary information

Supplementary Information

Reporting Summary

## Data Availability

The data used in this analysis are available from the National Geoscience Data Centre (NGDC) at the following link: 10.5285/cb950f2f-4c79-4ea8-ba9a-92a6dcc4f388^18^.
