# Supplementary material for: Comparative performance of rural water supplies during drought
Source: Nat Commun. 2020 Mar 4;11:1099. doi: 10.1038/s41467-020-14839-3 (PMC7055361; doi:10.1038/s41467-020-14839-3)
Supplement: Supplementary file 1 — Supplementary Information [file 41467_2020_14839_MOESM1_ESM.pdf]

Supplementary Information

Comparative performance of rural water supplies during drought

MacAllister et al.

Supplementary Figures

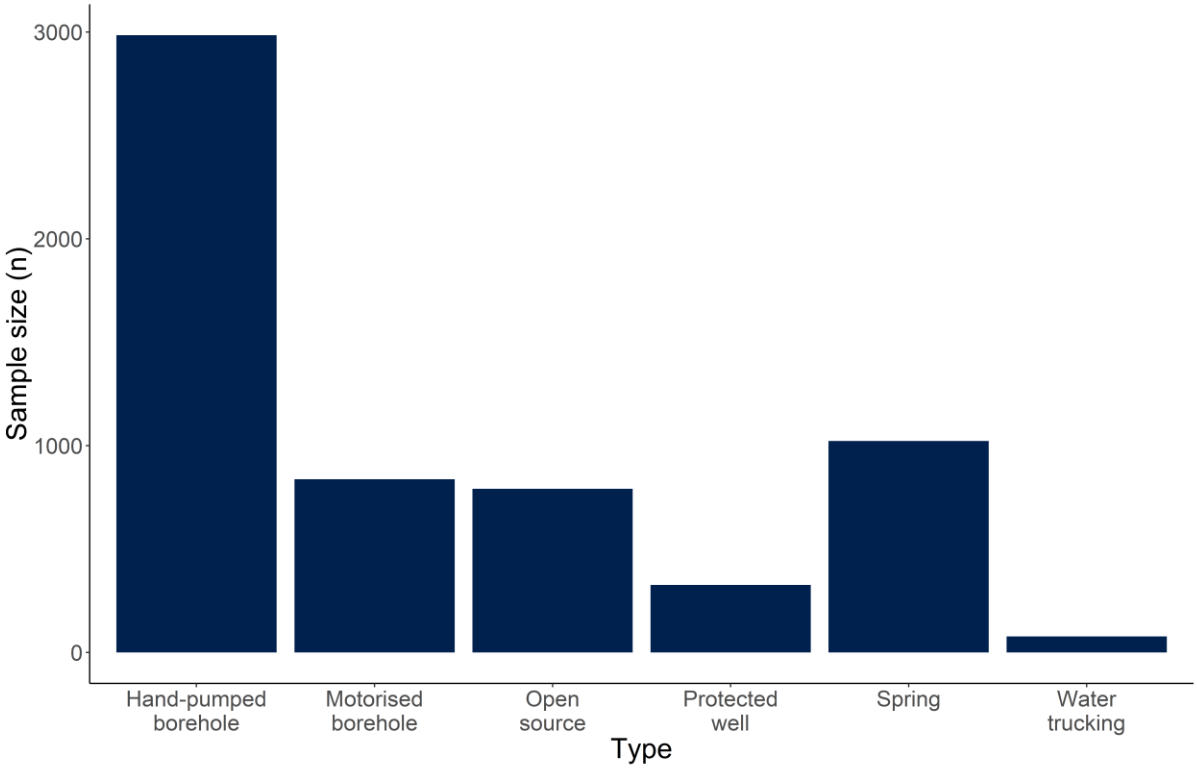

Figure 1 – Overall numbers of each water source type contained within the dataset and visited during the monitoring period.

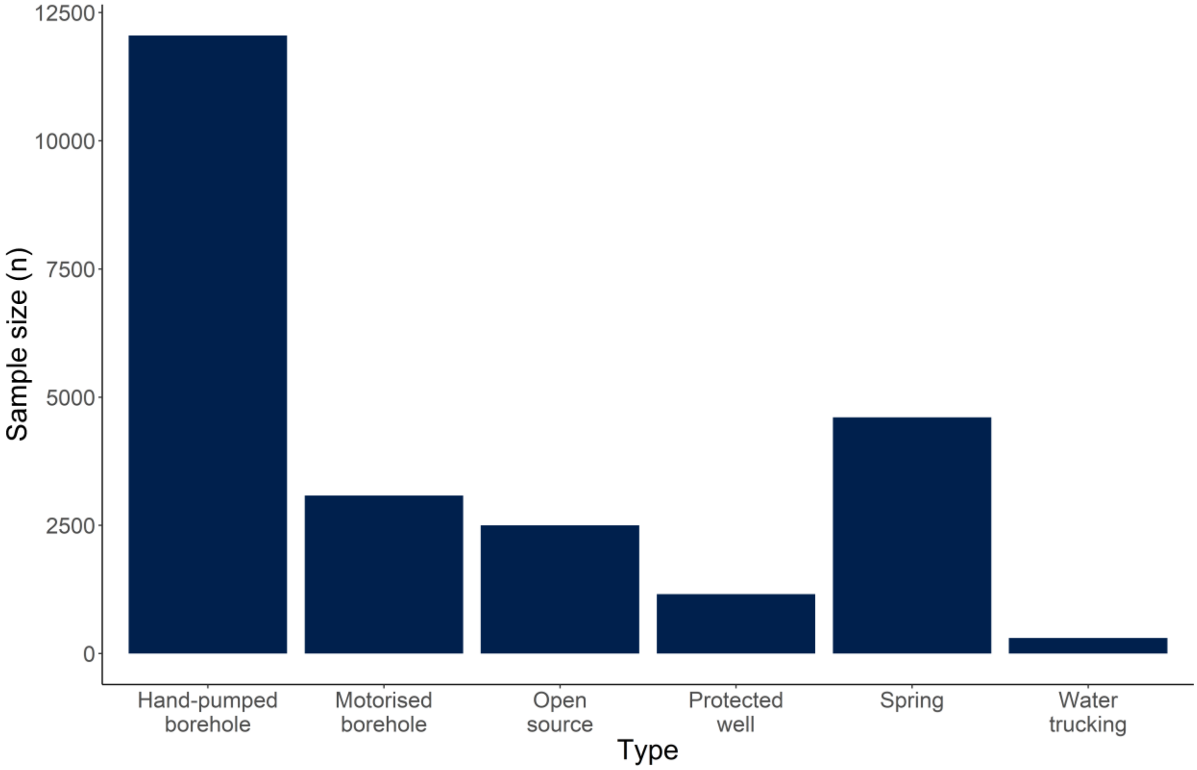

Figure 2 – Total number of site visits conducted for each water source type during the monitoring period.

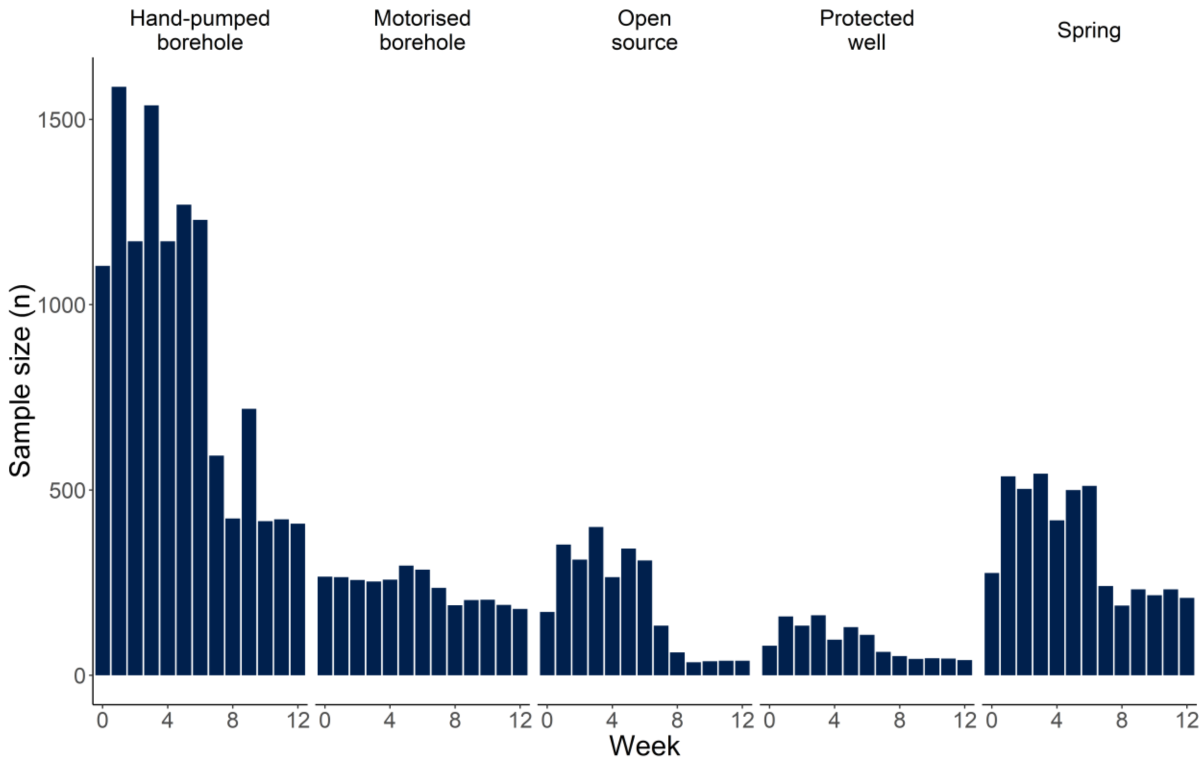

Figure 3 – Number of sites used to derive weekly functionality rates for each water source type during the monitoring period.

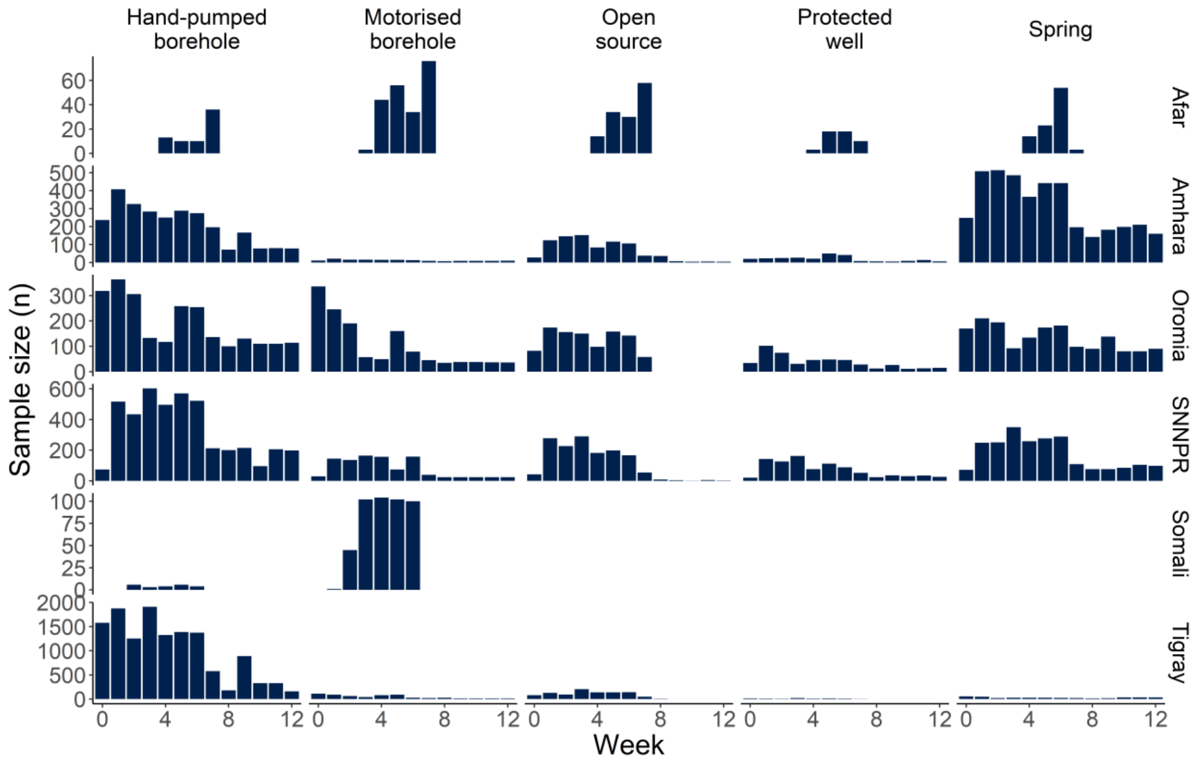

Figure 4 – Number of sites used to derive weekly functionality rates for each administrative region and water source type during the monitoring period.

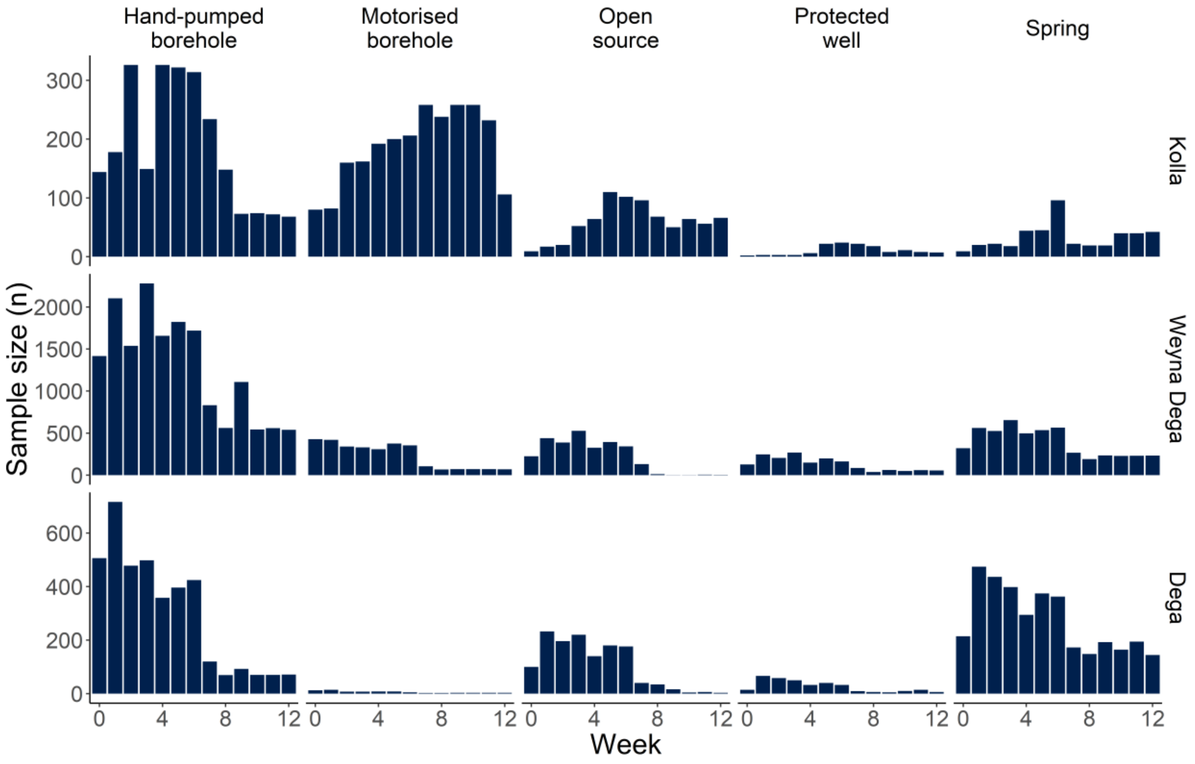

Figure 5 – Number of sites used to derive weekly functionality rates for each altitude zone and water source type during the monitoring period.

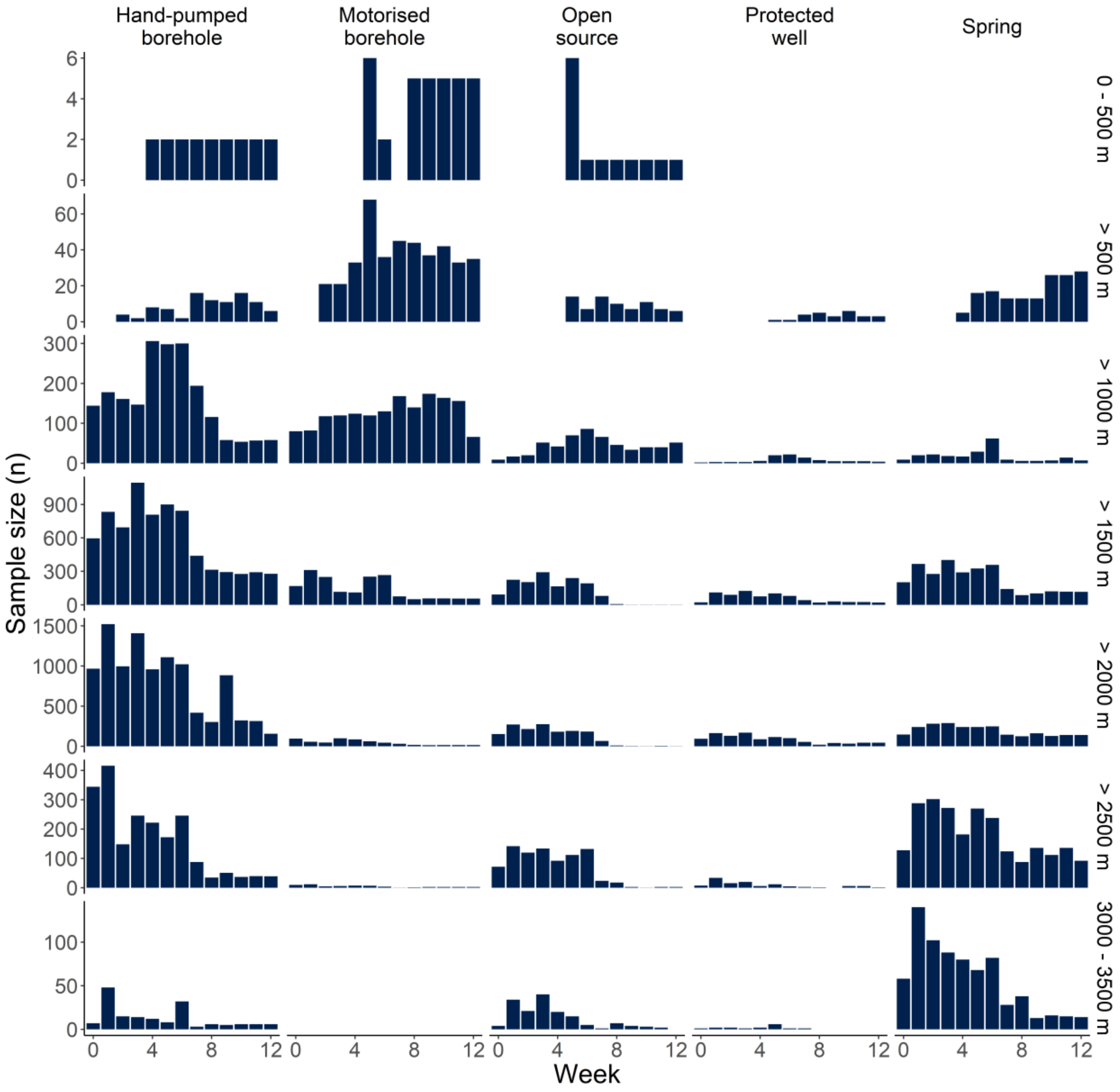

Figure 6 – Number of sites visited within 500 m altitude bins during the monitoring period.

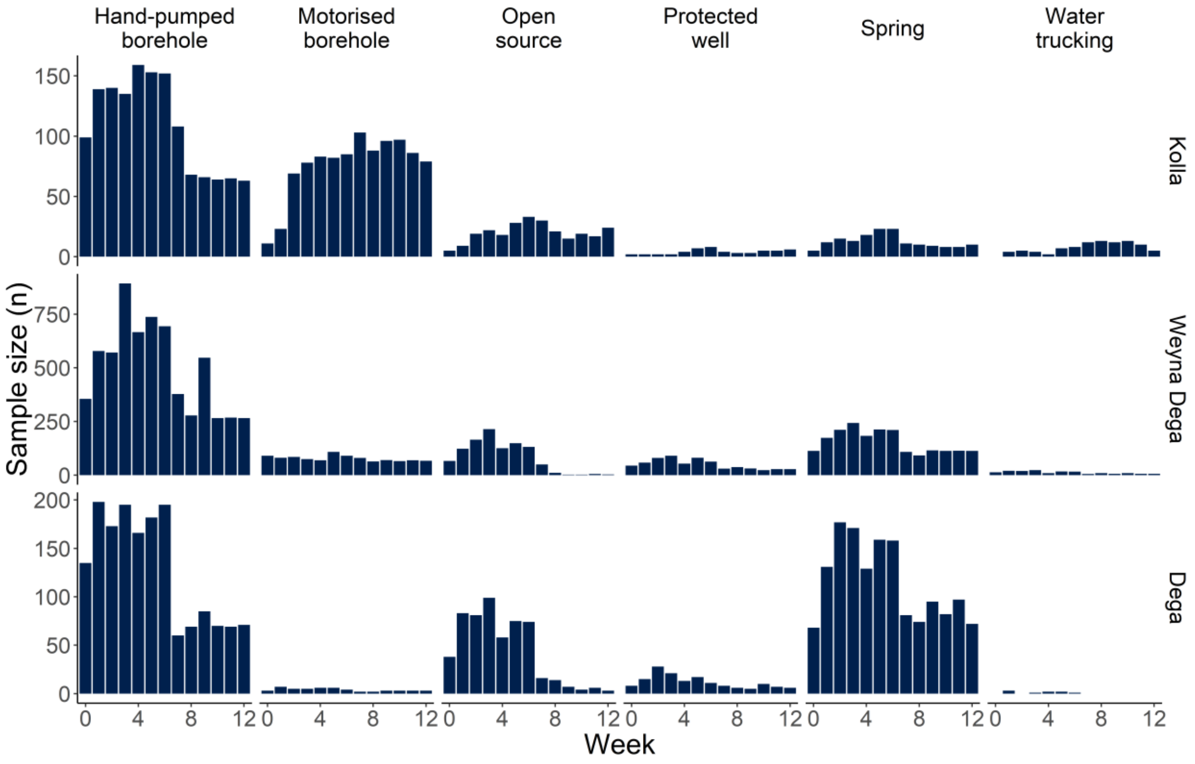

Figure 7 – Number of sites with estimates of user numbers for each altitude zone and water source type during the monitoring period.

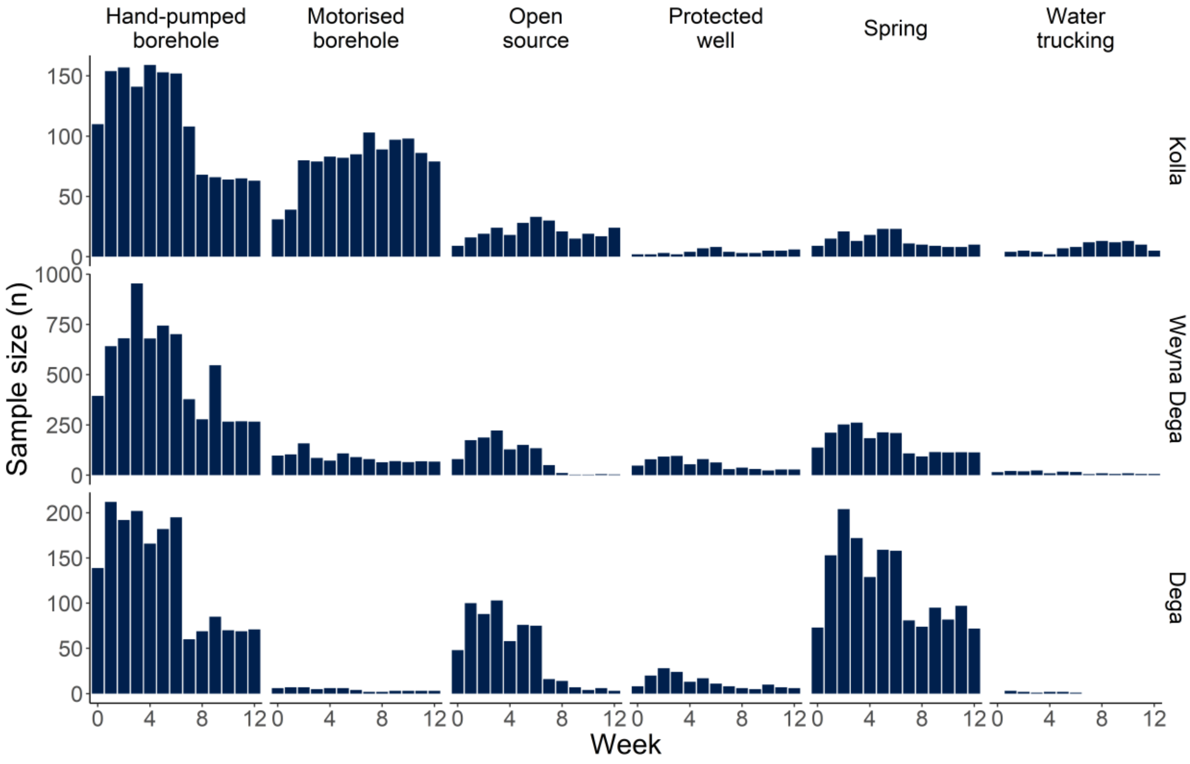

Figure 8 – Number of sites with the modal reported values of travel times for each altitude zone and water source type during the monitoring period.

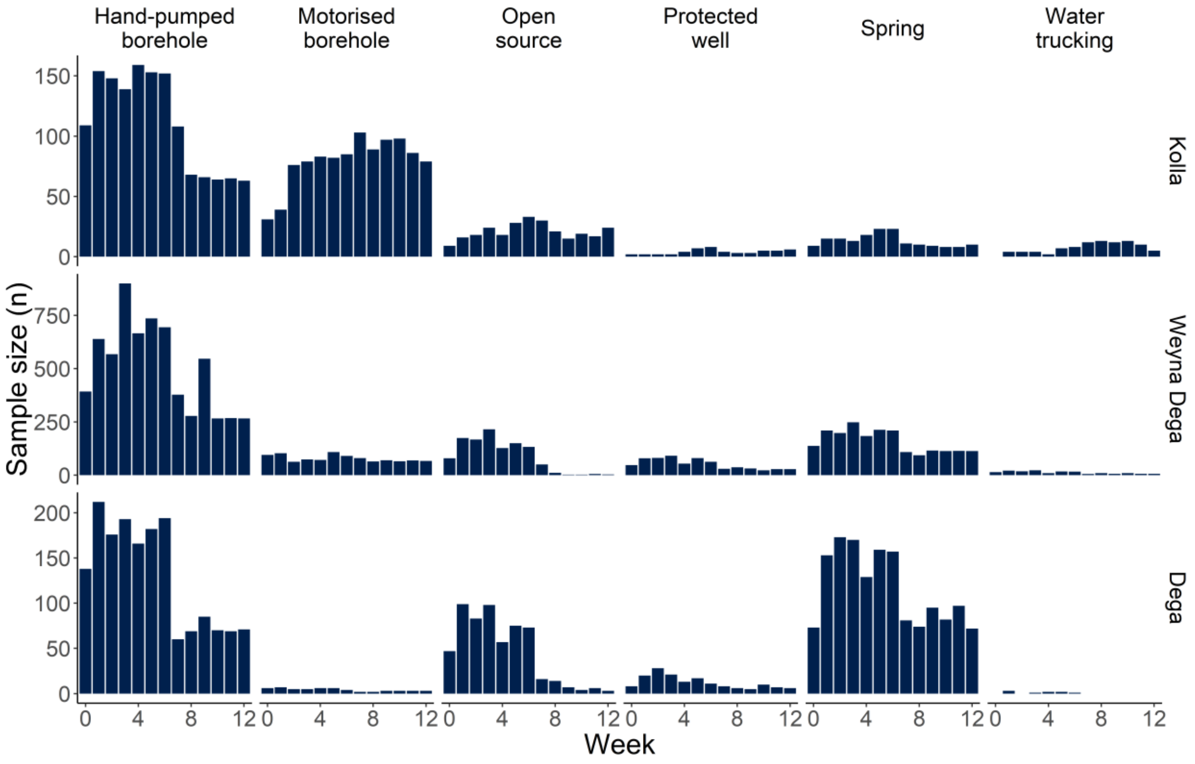

Figure 9 – Number of sites with reported user perception of the adequacy of water collected for each altitude zone and water source type during the monitoring period.

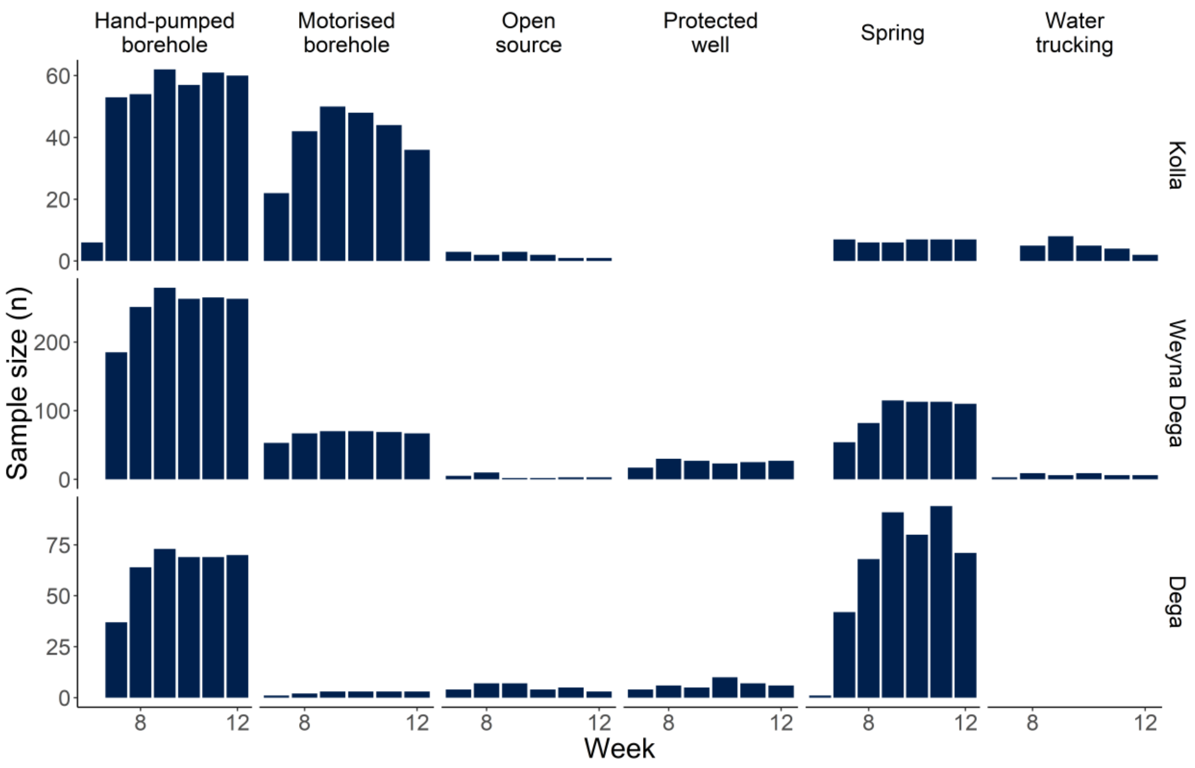

Figure 10 – Number of sites with the modal reported values of water quantities collected for each altitude zone and water source type during the monitoring period.

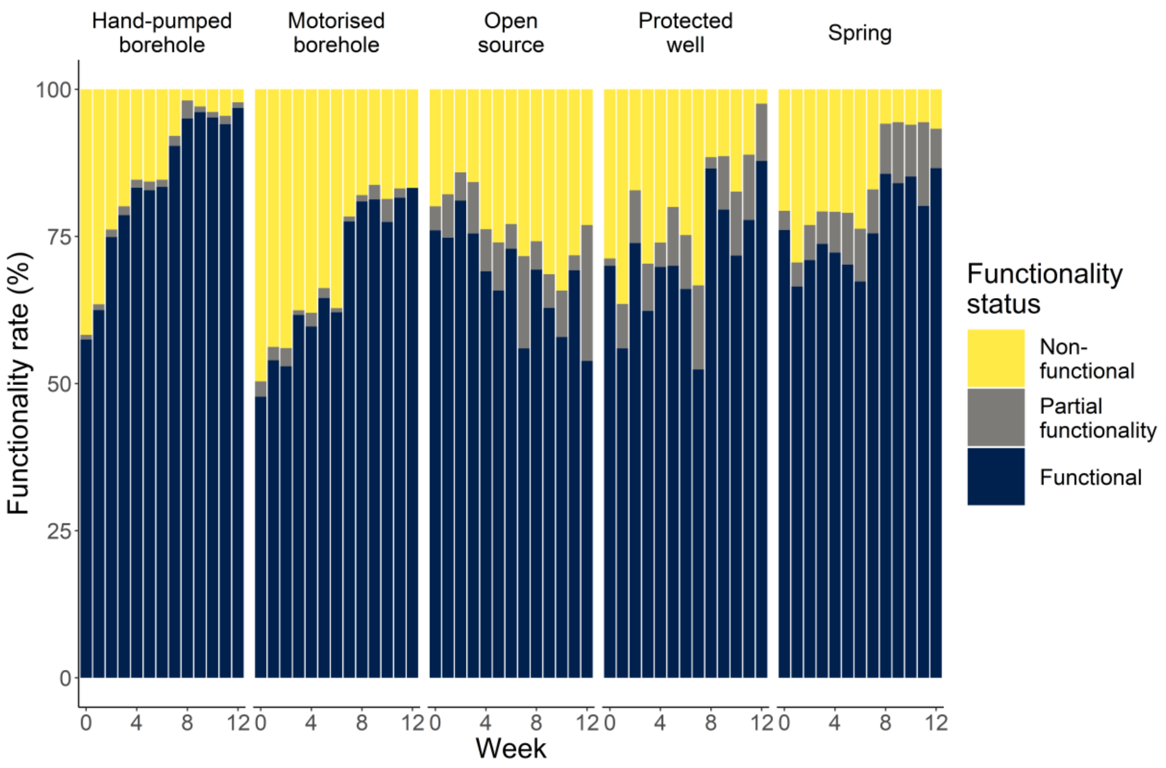

Figure 11 - Weekly functionality rate for each water source type during the monitoring period.

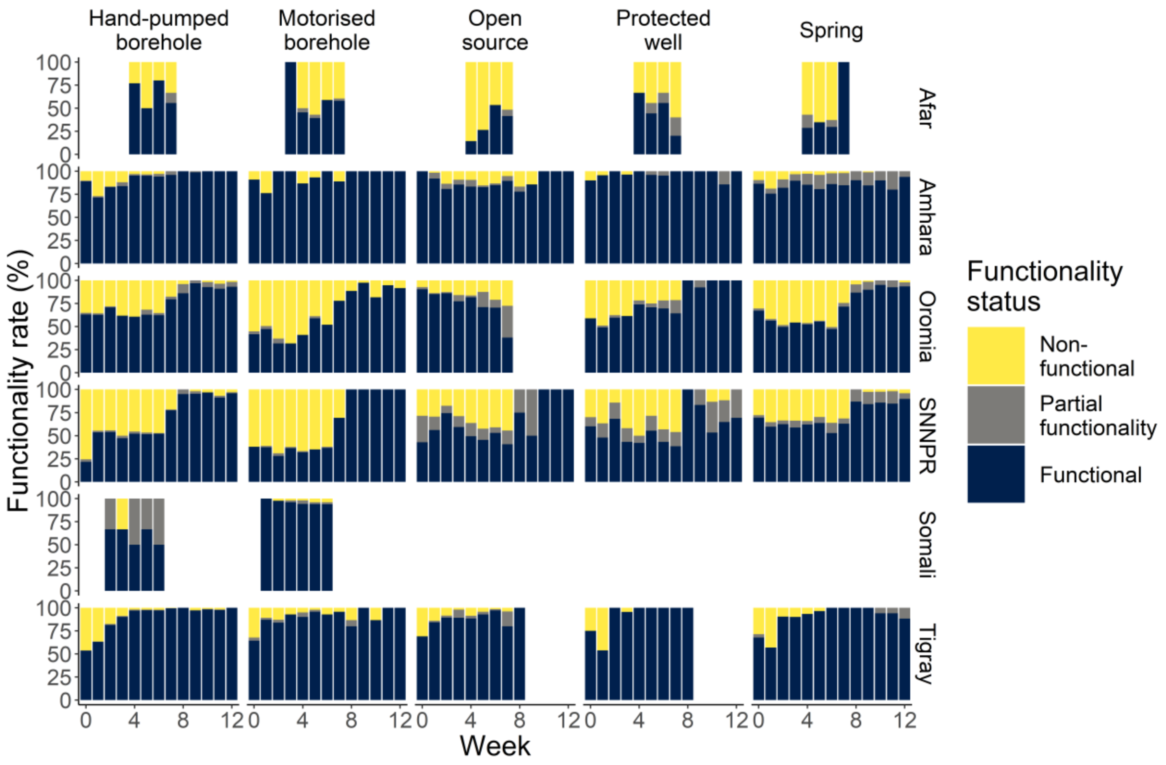

Figure 12 – Weekly functionality rates for each administrative region and each water source type during the monitoring period.

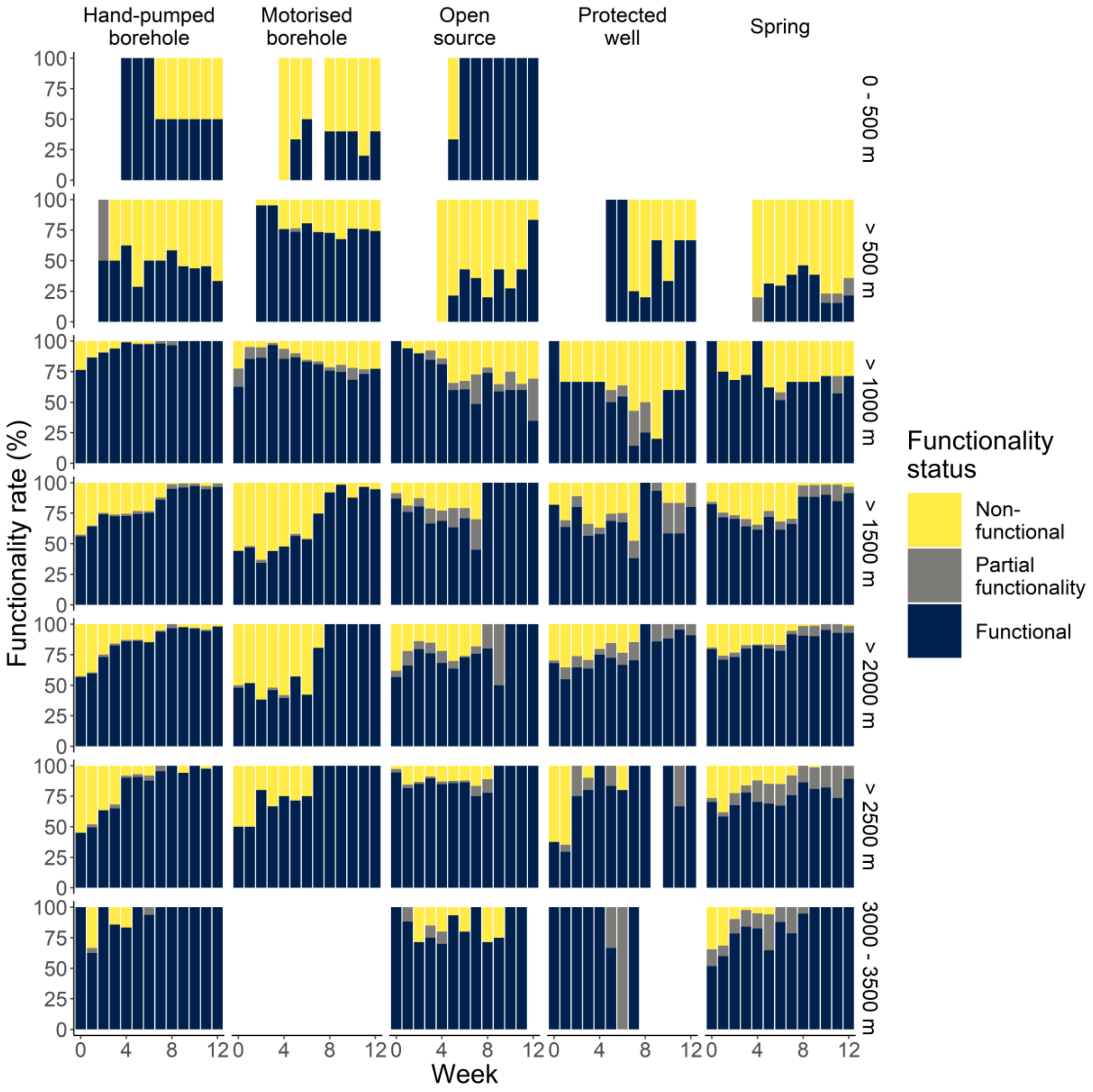

Figure 13 – Weekly functionality rates in 500 m altitude bins for each water source type during the monitoring period.

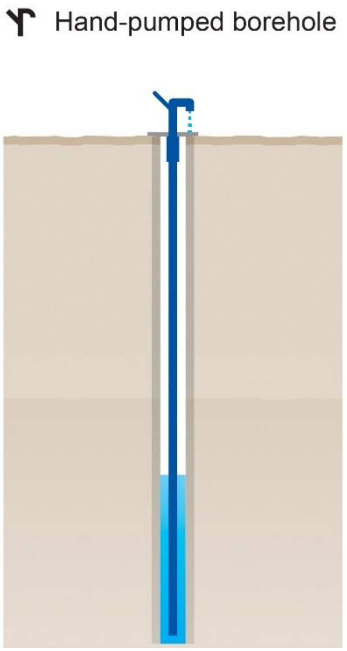

Figure 14 – Basic schematic of a hand-pumped borehole, see supplementary note 1section 1 for a detailed description of this water source type.

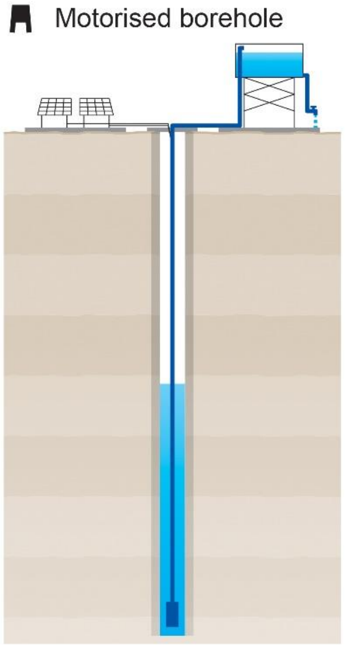

Figure 15 – Basic schematic of a motorised borehole with solar submersible pump, see supplementary note 1 section 2 for a detailed description of this water source type.

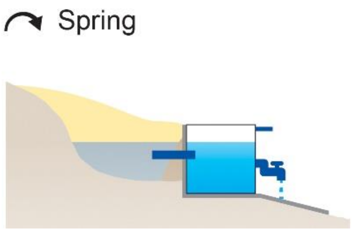

Figure 16 – Basic schematic of a protected spring with tap, see supplementary note 1 section 3 for a detailed description of this water source type.

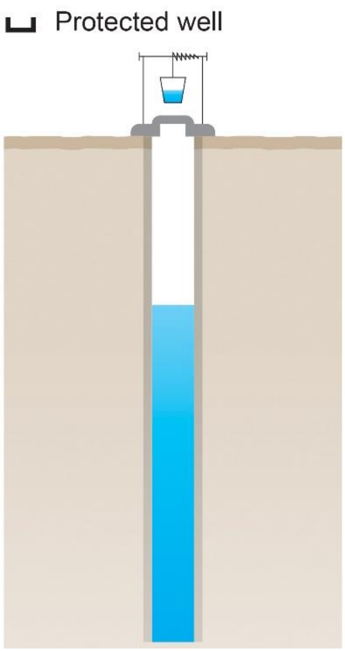

Figure 17 – Basic schematic of a protected hand-dug-well with bucket and windlass, see supplementary note 1 section 4 for a detailed description of this water source type.

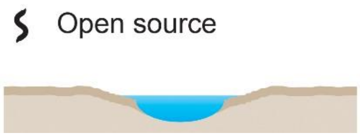

Figure 18 – Basic schematic of untreated surface water, see supplementary note 1 section 5 for a detailed description of this water source type.

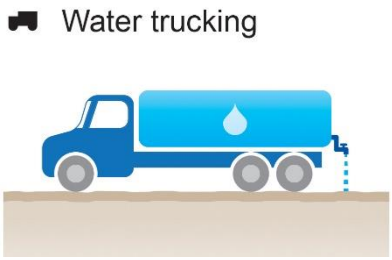

Figure 19 – Basic schematic of an emergency water truck, see supplementary note 1 section 6 for a detailed description of this water source type.

Supplementary Tables

Table 1 – Priority regions and Woredas where the UNICEF monitoring program took place.

Region/Monitoring agency	Woreda	Population	Altitude zone
**Tigray**<br>**World Vision Ethiopia**	Alamata	85,359	Weyna Dega
	Enderta	114,277	Weyna Dega
	Kilte Awlalo	99,688	Dega
	Seharti Samre	124,499	Weyna Dega
	Saesi Tsadamba	138,043	Dega
**Amhara**<br>**World Vision Ethiopia**	Ambasel	121,100	Dega
	Efrata Gidim	110,462	Weyna Dega
	Habru	192,701	Kolla
	Jile Timuga	72,864	Kolla
	Menz Gera Midir	102,420	Dega
**Oromia**<br>**World Vision Ethiopia**	Habro	191,546	Weyna Dega
	Jarso	119,368	Weyna Dega
	Jeju	124,046	Weyna Dega
	Melka Belo	176,813	Weyna Dega
	Shashemene Zuria	248,022	Weyna Dega
**SNNPR**<br>**World Vision Ethiopia**	Kochire	131,418	Weyna Dega
	Deniboya	81,771	Weyna Dega
	Kacha Bira	115,549	Weyna Dega
	Kedida Gamela	90,197	Weyna Dega
	Shashago	102,464	Weyna Dega
**Afar**<br>**Oxfam**	Abala	37,943	Kolla
	Berahile	78,850	Kolla
	Erebti	36,233	Kolla
	Megale	28,106	Kolla
**Somali**<br>**Oxfam**	Afdem	64,773	Kolla
	Ayisha	58,064	Kolla
	Denbel	82,040	Kolla
	Erer	77,544	Kolla
	Shinile	102,516	Kolla
	Mulo	71,497	Kolla
	**MONITORING AREA TOTAL**	**3,180,173**	

Supplementary Notes

Note 1: Water source types descriptions

1. Hand-pumps – Supplementary Figure 14

Hand-pumps are generally installed on boreholes between 15 and 45 m deep. Some hand pump designs are capable of pumping water from up to 100 m. Deep groundwater (>30 m) is generally more resilient to drought than shallower groundwater [15]. In Ethiopia the Afridev is the common type of hand-pump. Deep well versions of the India Mark II hand-pump are also becoming common in Ethiopia.

The most regular maintenance tasks required for hand-pumps are greasing of the bearings and inspection of the general condition of the pump. More significant (but less frequent) maintenance tasks include rethreading or replacing pump rods and rising mains. These more complex tasks usually require external support, particularly for some hand-pump models which need specialist equipment to remove the rising main and cylinder, including the India Mark II. Seals and bearings are highly vulnerable to wear and need replaced regularly. During drought it may be necessary to extend the rising main and rods, assuming the borehole is of sufficient depth to do so. All sources should be protected from contamination by maintaining the sanitary apron and fencing off the area around the source.

2. Motorised boreholes – Supplementary Figure 15

Motorised boreholes are particularly effective for accessing groundwater from depths exceeding the range of hand-pumps (i.e. 45 m - 100 m) but can also be used in areas with shallower groundwater. Motorised boreholes may also be appropriate in areas where aquifers are able to sustain high yields and large pumping rates (rates exceeding 1 litre per second). Motorised boreholes usually rely on a diesel generator or solar powered submersible pump. With current technology diesel pumps can pump from depths of up to 600 m, whereas solar pumps can pump from depths of up to 250 m. Motorised boreholes rely on centrifugal pumps placed above ground or submersible pumps placed under water. Multistage centrifugal pumps are required when pumping from depths greater than 50m.

Motorised boreholes must be maintained by a trained caretaker and require effective external support. Diesel powered pumps require refuelling on a regular basis. Pump oil and water levels should be monitored and replaced when necessary. In some situations fuel might be expensive or scare affecting on-going operation. At regular intervals filters should be cleaned, oil changed and components such as nuts, bolts and the exhaust replaced. Solar powered pumps require regular maintenance of solar panels, including checking for damage and cleaning.

The most frequent problems with motorised boreholes is excessive wear of the moving components and, in the case of electrical and solar pumps, failure of wiring and electrical components. Submersible pumps may also fail if there is excessive silt or if the borehole runs dry. Thus, the pump

should be placed at an appropriate level below the water table and removed regularly to clear silt. Groundwater levels should be monitored routinely when using submersible pumps to ensure that the risk of the submersible pump running dry is minimised. Pipes associated with the distribution system are often reported as a cause of failure for motorised boreholes and, along with storage tanks, require regular inspection. In general maintenance and repair is more complicated than that required for hand-pumps and generally requires a higher level of skill and external expertise. For both hand-pumped and motorised boreholes it is essential that spare parts are readily available.

3. Springs – Supplementary Figure 16

Protected springs and springs providing gravity fed water supplies receive water through natural groundwater flow and are common in mountainous areas or areas with undulating topography. Springs arise when the water table intersects the land surface. This may be due to changes in rock type, faults which intersect the surface or breaks in slope. The water flowing from springs has a certain residence time in the sub-surface. For example, springs arising as a result of a break of slope may have a low residence time and tap shallow aquifer systems. These springs may be intermittent and/or more vulnerable to failure during the dry season or drought. Conversely, springs tapping fault systems are likely to have longer residence times and tap much deeper, and more resilient, groundwater systems.

Protected springs consist of an evacuated area backfilled with sand and clay layers around the eye of the spring. The backfill layers prevent leakage of contaminated surface water into the eye of the spring. A pipe is installed in the most permeable area of the backfill. The pipe leads to a spring box where water is stored. The water can then be collected from a tap in the spring box or distributed via a gravity piped system. The most common maintenance tasks for protected springs include ensuring that water is able to flow freely from the spring box and into the drainage system, and ensuring that the area surrounding the spring is clean. If springs become blocked, the protective backfill layers may need to be replaced. Other tasks may include silt removal and leak repair. Most operation and maintenance tasks can be carried out without external support. When protected springs feed gravity water supplies, regular inspection of the pipe network is required to ensure that there is no leakage. Pipes may need to be repaired and the taps on stand posts may need to be replaced.

4. Protected wells – Supplementary Figure 17

Protected hand-dug-wells have a much larger diameter than boreholes. Diameters range from a minimum of 0.8 m up to several metres. Hand-dug-wells should be lined, they usually have a wall surrounding the well at the surface and a protective cover. Well depths are relatively shallow (compared with boreholes), but can range from a few meters to over 50 m in some cases. The larger diameter of hand-dug-wells means that, unlike boreholes, they can provide some water storage. Protected hand-dug-wells may be fitted with a rope and bucket (sometimes on a pulley or a windlass),

a hand-pump, or even a motorised pump. Typical maintenance tasks for protected hand-dug-wells include repair of protective coverings and linings, usually by a specialist mason. If water levels fall wells can be deepened, but this is a much harder, and more time consuming, task than extending the pumped depth of hand-pumped or motorised boreholes.

5. Open sources – Supplementary Figure 18

Open sources include surface water without treatment, unprotected hand-dug-wells and unprotected springs. All of these sources are classified as unimproved. Surface water is highly vulnerable to contamination and is more likely to dry up during drought.

6. Water trucking – Supplementary Figure 19

Emergency water trucking involves transporting water from another area or catchment into a drought-affected area for temporary storage or immediate distribution. Water may need to be treated prior to distribution if drawn from vulnerable sources. Emergency water trucking is a high cost drought intervention and typically an intervention of last resort.

Note 2: Overview of the role of mobile maintenance teams

During the period from January to May 2016 UNICEF contracted mobile maintenance teams in four strategic areas to support the Water, Sanitation and Hygiene Committees (WASHCo) in operation and maintenance of hand-pumped and motorised boreholes and electromechanical equipment. The mobile teams were expected to ensure sufficient stock of spare parts supply for electromechanical equipment. An overview of the roles and responsibility of the maintenance teams is provided below.

1. Objective

- To sustain water supply provision from existing boreholes by preventing failure in drought affected Woredas.

2. Tasks

- Conduct a rapid assessment of selected motorised borehole schemes and identify and itemise the following; type of generator, control board and other electrical equipment. Gather all other relevant information.
- Evaluate conceptual designs of electromechanical equipment installation.
- Support WASHCo's in correct installation of cables, switchboards and generators to ensure stability of electrical supply during power fluctuations.
- Test and maintain electric switchboard control panels, generator and all required cables.
- Maintain/replace all components found to be faulty or at end of serviceable life.
- Develop fault indicators and troubleshooting procedures, easily understood by WASHCo’s and operators, on the following: (i) Pumps, (ii) Generators, (iii) Other electromechanical equipment.
- Assist WASHCo’s in developing daily maintenance checklists for scheduled maintenance.
- Ensure teams are equipped with the required tools and spare parts at all times.
- Assist in identifying appropriate and high quality spare parts and preparation of technical specifications of spare parts required for operation and maintenance of equipment.
- Train WASHCo's and equipment operators in all procedures required to test and maintain electromechanical equipment to ensure water supply remains functional.
- Ensure transfer of knowledge and skills to WASHCo’s and equipment operators for on-going operation and maintenance of electromechanical equipment.
- Support development and implementation of regional workshop focusing on operation and maintenance of electromechanical equipment.
- Support the development of a protocol for testing functionality of all equipment.
- Submit a monthly report to RWB including the list of activities performed and list of all electromechanical equipment.
