# Supplementary material for: Comparative performance of rural water supplies during drought
Source: Nat Commun. 2020 Mar 4;11:1099. doi: 10.1038/s41467-020-14839-3 (PMC7055361; doi:10.1038/s41467-020-14839-3)
Supplement: Supplementary file 2 — Reporting Summary [file 41467_2020_14839_MOESM2_ESM.pdf]

nature research

Corresponding author(s): *Double-blind peer review submissions: write DBPR and your manuscript number here instead of author names.*

Last updated by author(s): *YYYY-MM-DD*

Reporting Summary

Nature Research wishes to improve the reproducibility of the work that we publish. This form provides structure for consistency and transparency in reporting. For further information on Nature Research policies, see Authors & Referees and the Editorial Policy Checklist.

Statistics

For all statistical analyses, confirm that the following items are present in the figure legend, table legend, main text, or Methods section.

n/a	Confirmed	
☒	☐	The exact sample size (*n*) for each experimental group/condition, given as a discrete number and unit of measurement
☒	☐	A statement on whether measurements were taken from distinct samples or whether the same sample was measured repeatedly
☒	☐	The statistical test(s) used AND whether they are one- or two-sided<br>*Only common tests should be described solely by name; describe more complex techniques in the Methods section.*
☒	☐	A description of all covariates tested
☒	☐	A description of any assumptions or corrections, such as tests of normality and adjustment for multiple comparisons
☒	☐	A full description of the statistical parameters including central tendency (e.g. means) or other basic estimates (e.g. regression coefficient) AND variation (e.g. standard deviation) or associated estimates of uncertainty (e.g. confidence intervals)
☒	☐	For null hypothesis testing, the test statistic (e.g. *F*, *t*, *r*) with confidence intervals, effect sizes, degrees of freedom and *P* value noted<br>*Give P values as exact values whenever suitable.*
☒	☐	For Bayesian analysis, information on the choice of priors and Markov chain Monte Carlo settings
☒	☐	For hierarchical and complex designs, identification of the appropriate level for tests and full reporting of outcomes
☒	☐	Estimates of effect sizes (e.g. Cohen's *d*, Pearson's *r*), indicating how they were calculated

Our web collection on statistics for biologists contains articles on many of the points above.

Software and code

Policy information about availability of computer code

Data collection	*NA*
Data analysis	*NA*

For manuscripts utilizing custom algorithms or software that are central to the research but not yet described in published literature, software must be made available to editors/reviewers. We strongly encourage code deposition in a community repository (e.g. GitHub). See the Nature Research guidelines for submitting code & software for further information.

Data

Policy information about availability of data

All manuscripts must include a data availability statement. This statement should provide the following information, where applicable:

- Accession codes, unique identifiers, or web links for publicly available datasets
- A list of figures that have associated raw data
- A description of any restrictions on data availability

The data used in this analysis is available from the National Geoscience Data Centre (NGDC) at the following link: https://dx.doi.org/10.5285/cb950f2f-4c79-4ea8-ba9a-92a6dcc4f388.

Field-specific reporting

Please select the one below that is the best fit for your research. If you are not sure, read the appropriate sections before making your selection.

☐ Life sciences ☐ Behavioural & social sciences ☒ Ecological, evolutionary & environmental sciences

Life sciences study design

All studies must disclose on these points even when the disclosure is negative.

Sample size	*NA*
Data exclusions	*NA*
Replication	*NA*
Randomization	*NA*
Blinding	*NA*

Behavioural & social sciences study design

All studies must disclose on these points even when the disclosure is negative.

Study description	*NA*
Research sample	*NA*
Sampling strategy	*NA*
Data collection	*NA*
Timing	*NA*
Data exclusions	*NA*
Non-participation	*NA*
Randomization	*NA*

Ecological, evolutionary & environmental sciences study design

All studies must disclose on these points even when the disclosure is negative.

Study description

Ethiopia experienced severe drought in 2015-16. Many rural communities experienced declines in the performance of their water supply systems. As a result UNICEF commissioned a real-time monitoring and responsive operation and maintenance programme for point source rural water supplies across Central, Northern and Eastern Ethiopia. The water point monitoring survey was coordinated by UNICEF and conducted by World Vision Ethiopia and Oxfam Ethiopia. Data was collected between January and May 2016. Akvo Flow, a mobile survey tool, was used to collect data using questionnaires which were completed by enumerators and uploaded to central servers in near real time. The dataset includes data on functionality, access, usage and water quantity from 5196 rural water points. UNICEF provided the dataset to BGS. BGS reorganised, cleaned, and conducted quality control and analysis of the dataset.

Research sample

NA

Sampling strategy

Water source monitoring data were collected from January to May 2016 by teams commissioned by UNICEF following the severe drought in Ethiopia in late 2015 and early 2016. The monitoring programme was conducted in three phases and collected data on source functionality, usage and access. Figure 1(a) shows the locations of individual water points surveyed during the monitoring programme. The first phase of the programme comprised of a baseline inventory survey, involving a census-style asset tagging exercise of all water points in the target Woredas (administrative divisions' equivalent to districts). Details of the Woredas included in the surveys can be found in the supplementary information (Supplementary Table 1). During the census phase, 5196 individual sites were visited. The second phase consisted of a monitoring survey performed weekly on a sub-sample of water points. A total of 3188 sites were visited more than once during the monitoring survey. The mean number of visits per site for the 12 weeks of the survey was 3.5. The total numbers of each water source type visited each week and the total number of sites visited per week in each administrative region are shown in Supplementary Figure 3 and 4 respectively. The final phase, which was only conducted for the last month of the monitoring programme, consisted of a household survey to establish the quantities of water collected by users during the drought.

Data collection

For each phase trained enumerators used questionnaires to gather quantitative and qualitative data. Surveys were conducted by questioning water point attendants, a water, sanitation and hygiene (WASH) committee member or another knowledgeable local authority. The surveys were conducted using a mobile monitoring platform called Akvo Flow which is developed by The Akvo

Foundation. The Akvo Flow application was installed on Samsung Galaxy J1 smartphones (model number SM-J100H) which used Android version 4.4.4. Digital survey forms were pre-loaded onto the app. All data were stored on the smartphones and automatically sent to the Akvo database whenever an internet connection was available.

Timing and spatial scale: *NA*

Data exclusions: *NA*

Reproducibility: *NA*

Randomization: *NA*

Blinding: *NA*

Did the study involve field work? ☒ Yes ☐ No

Field work, collection and transport

Field conditions: *During drought in Ethiopia: 26/01/2016 to 24/04/2016*

Location: *Ethiopia bounding coordinates (WGS84): 37.716, 7.170; 42.867, 9.896*

Access and import/export: *NA*

Disturbance: *NA*

Reporting for specific materials, systems and methods

We require information from authors about some types of materials, experimental systems and methods used in many studies. Here, indicate whether each material, system or method listed is relevant to your study. If you are not sure if a list item applies to your research, read the appropriate section before selecting a response.

Materials & experimental systems

n/a	Involved in the study
☒	☐ Antibodies
☒	☐ Eukaryotic cell lines
☒	☐ Palaeontology
☒	☐ Animals and other organisms
☒	☐ Human research participants
☒	☐ Clinical data

Methods

n/a	Involved in the study
☒	☐ ChIP-seq
☒	☐ Flow cytometry
☒	☐ MRI-based neuroimaging

Antibodies

Antibodies used: *NA*

Validation: *NA*

Eukaryotic cell lines

Policy information about cell lines

Cell line source(s): *NA*

Authentication: *NA*

Mycoplasma contamination: *NA*

Commonly misidentified lines (See ICLAC register): *NA*

Palaeontology

Specimen provenance: *NA*

Specimen deposition: *NA*

Dating methods	*NA*

☐ Tick this box to confirm that the raw and calibrated dates are available in the paper or in Supplementary Information.

Animals and other organisms

Policy information about studies involving animals; ARRIVE guidelines recommended for reporting animal research

Laboratory animals	*NA*
Wild animals	*NA*
Field-collected samples	*NA*
Ethics oversight	*NA*

Note that full information on the approval of the study protocol must also be provided in the manuscript.

Human research participants

Policy information about studies involving human research participants

Population characteristics	*NA*
Recruitment	*NA*
Ethics oversight	*NA*

Note that full information on the approval of the study protocol must also be provided in the manuscript.

Clinical data

Policy information about clinical studies

All manuscripts should comply with the ICMJE guidelines for publication of clinical research and a completed CONSORT checklist must be included with all submissions.

Clinical trial registration	*NA*
Study protocol	*NA*
Data collection	*NA*
Outcomes	*NA*

ChIP-seq

Data deposition

☐ Confirm that both raw and final processed data have been deposited in a public database such as GEO.

☐ Confirm that you have deposited or provided access to graph files (e.g. BED files) for the called peaks.

Data access links<br>*May remain private before publication.*	*NA*
Files in database submission	*NA*
Genome browser session<br>(e.g. UCSC)	*NA*

Methodology

Replicates	*NA*
Sequencing depth	*NA*
Antibodies	*NA*
Peak calling parameters	*NA*
Data quality	*NA*

Software | *NA*

Flow Cytometry

Plots

Confirm that:

☐ The axis labels state the marker and fluorochrome used (e.g. CD4-FITC).

☐ The axis scales are clearly visible. Include numbers along axes only for bottom left plot of group (a 'group' is an analysis of identical markers).

☐ All plots are contour plots with outliers or pseudocolor plots.

☐ A numerical value for number of cells or percentage (with statistics) is provided.

Methodology

Sample preparation | *NA*

Instrument | *NA*

Software | *NA*

Cell population abundance | *NA*

Gating strategy | *NA*

☐ Tick this box to confirm that a figure exemplifying the gating strategy is provided in the Supplementary Information.

Magnetic resonance imaging

Experimental design

Design type | *NA*

Design specifications | *NA*

Behavioral performance measures | *NA*

Acquisition

Imaging type(s) | *NA*

Field strength | *NA*

Sequence & imaging parameters | *NA*

Area of acquisition | *NA*

Diffusion MRI ☐ Used ☐ Not used

Preprocessing

Preprocessing software | *NA*

Normalization | *NA*

Normalization template | *NA*

Noise and artifact removal | *NA*

Volume censoring | *Define your software and/or method and criteria for volume censoring, and state the extent of such censoring.*

Statistical modeling & inference

Model type and settings | *NA*

Effect(s) tested | *NA*

Specify type of analysis: ☐ Whole brain ☐ ROI-based ☐ Both

Statistic type for inference
(See Eklund et al. 2016)

NA

Correction

NA

Models & analysis

n/a	Involved in the study
☒	☐ Functional and/or effective connectivity
☒	☐ Graph analysis
☒	☐ Multivariate modeling or predictive analysis

Functional and/or effective connectivity

NA

Graph analysis

NA

Multivariate modeling and predictive analysis

NA
